# A dual-function XCL1-CD40L mRNA adjuvant potentiates cancer vaccines by coordinating DC recruitment and maturation

**DOI:** 10.3389/fimmu.2026.1875603

**Published:** 2026-10-14

**Authors:** Chunjie Wu, Wanxuan Shao, Yadi Liu, Shuyuan Pan, Wei Wang, Li Deng, Suyue Zhang, Zhimin Cui, Yujiao Wang, Peng He, Yong Yang, Yiheng Zhao

**Affiliations:** 1 School of Basic Medicine and Clinical Pharmacy, China Pharmaceutical University, Nanjing, China; 2 Department of Research and Development, Beijing Institute of Biological Products Co., Ltd, Beijing, China; 3 State Key Laboratory of Novel Vaccines for Emerging Infectious Diseases, Beijing, China; 4 School of Pharmacy, China Pharmaceutical University, Nanjing, China; 5 Center for New Drug Safety Evaluation and Research, State Key Laboratory of Natural Medicines, China Pharmaceutical University, Nanjing, China

**Keywords:** cancer immunotherapy, cDC1, mRNA adjuvant, mRNA vaccine, tumor microenvironment, XCL1-CD40L

## Abstract

**Background:**

Despite the significant promise of mRNA tumor vaccines in treating solid tumors, their clinical response rate is constrained by the low effectiveness of antigen presentation resulting from the immune-suppressive tumor microenvironment (TME). Conventional type 1 dendritic cells (cDC1) are pivotal in antigen cross-presentation; yet, they frequently exhibit insufficient immune-activating communication within solid malignancies.

**Methods:**

This investigation comprehensively examined the functional attributes of cDC1 by TCGA cohorts and single-cell transcriptomic data. Guided by these bioinformatic insights, we engineered and synthesized a dual-functional mRNA adjuvant encoding the mXCL1-mCD40L fusion protein. We assessed the biological functions and anti-tumor efficacy of this adjuvant in combination with Glypican-3 (GPC3) and HPV-associated mRNA vaccines via protein expression, structure prediction, *in vivo* antigen uptake and presentation assays, antigen-presenting cell activation assays, antigen-specific T cell activation tests, and therapeutic studies in tumor-bearing mouse models.

**Results:**

A high cDC1 signature score was associated with improved overall survival across LIHC and SKCM TCGA cohorts. Compared to normal sample, cDC1s in HCC and melanoma retained their antigen-presentation machinery but received reduced activating ligand inputs (XCL1-XCR1 signaling in HCC and CD40-CD40L signaling in melanoma). AlphaFold 3 showed that the mXCL1-mCD40L fusion protein can maintain structural integrity without spatial steric hindrance. The fusion protein was confirmed to be secreted and to retain both XCL1-dependent chemotactic activity and CD40-dependent dendritic-cell activation. mXCL1-mCD40L mRNA-LNP activated macrophages and dendritic cells *in vitro*, upregulating immune-related gene expression as confirmed by qPCR. *In vivo*, the dual-functional adjuvant significantly enhanced the anti-tumor effect of the GPC3 vaccine, outperformed co-delivery of separate mXCL1 and mCD40L mRNA-LNP, and induced stronger specific IFN-γ producing T cells. Meanwhile, this adjuvant also boosted the anti-tumor efficacy of HPV-associated mRNA vaccines. Mechanistically, this adjuvant increased tumor-antigen acquisition and cross-presentation by CD103^+^ DCs and promoted DC maturation in draining lymph nodes, accompanied by an increased proportion of peripheral CD8^+^ T cells.

**Conclusion:**

By enhancing the recruitment and maturation of cDC1s, the dual-function mXCL1-mCD40L mRNA adjuvant boosts the efficacy of GPC3 and HPV-associated mRNA vaccines, supporting its potential as an antigen-flexible adjuvant platform against solid tumors.

## 1 Introduction

Glypican-3 (GPC3) is a membrane-associated sulfated heparan sulfate proteoglycan. It is minimally expressed in normal adult tissues but is prominently and specifically expressed in 70%-90% of hepatocellular carcinoma (HCC) (1). It is also aberrantly expressed in subgroups of refractory solid tumors, including lung cancer and melanoma (2–4). Furthermore, its expression level is markedly associated with malignant progression, invasion, metastasis, and poor prognosis of the tumor. It is acknowledged as a highly promising tumor-associated antigen in the domain of solid malignancies. At present, several antibody therapeutics directed against GPC3, CAR-T cell therapies, and peptide vaccines have entered clinical trials, validating the safety and anti-tumor potential of this target (5, 6). mRNA vaccines aimed at GPC3 have emerged as a focal point in the advancement of targeted therapies for HCC (7, 8).

Dendritic cells (DCs) are the most potent professional antigen-presenting cells (9). Among them, the conventional type 1 dendritic cells (cDC1) are the principal subset specialized in antigen cross-presentation, which is crucial for initiating CD8^+^ cytotoxic T cell-mediated anti-tumor immune responses (10). Numerous studies have confirmed that cDC1s frequently exhibit impaired functional states within the microenvironment of solid tumors. First, insufficient or dysregulated chemokine cues may limit optimal cDC1 positioning, retention, and interaction with tumor antigens and T cells (11, 12). Second, hypoxia and immunosuppressive networks within the tumor microenvironment (TME) can impair cDC1 maturation, downregulate co-stimulatory molecules, and compromise effective antigen presentation and T cell activation (13). These two defects jointly constitute the core barrier to the efficacy of mRNA vaccines and represent critical bottlenecks that existing adjuvants have failed to address simultaneously (14).

XCL1 is a member of the chemokine family, and its receptor XCR1 is selectively enriched on the surface of cDC1 (15). The XCL1-XCR1 axis is the key chemokine pathway regulating the recruitment of cDC1 in the body (11, 16). CD40L is a member of the tumor necrosis factor superfamily, which can activate the NF-κB pathway by binding to the CD40 on the surface of DCs and is a key co-stimulatory signal driving DC maturation and enhancing the ability of antigen cross-presentation (17, 18). Previous studies have explored the potential of XCL1 or CD40L as vaccine adjuvants (19–23). However, strategies based on the co-delivery of two independent mRNA molecules may face challenges such as asynchronous biodistribution, unmatched expression kinetics, and more complex formulation processes (24). Moreover, rational adjuvant design guided by single-cell evidence from clinical solid tumor samples remains insufficiently explored. Therefore, developing a dual-functional mRNA adjuvant that can simultaneously achieve specific recruitment and functional activation of cDC1 holds promising theoretical value for improving the anti-solid tumor efficacy of GPC3 mRNA vaccines.

Based on the above research status and unmet clinical needs, we hypothesized that simultaneous targeting of cDC1 chemotactic recruitment and functional maturation can enhance cDC1-associated immune activation in the solid TME, and significantly enhance the anti-tumor efficacy of mRNA vaccines. To test this hypothesis, we first integrated TCGA datasets and scRNA-seq datasets from different solid tumors (HCC and melanoma) to systematically dissect the functional abnormalities and underlying molecular mechanisms of cDC1 in the TME. We then designed and constructed a dual-functional mRNA adjuvant encoding the mXCL1-mCD40L fusion protein, and verified its immune activation activity *in vitro*. Furthermore, we evaluated the synergistic anti-tumor efficacy and underlying mechanism of this adjuvant in combination with GPC3 mRNA vaccine in tumor-bearing mouse models. Finally, we assessed the preliminary *in vivo* safety and formulation feasibility of this combination regimen, aiming to provide a promising technical strategy and experimental basis for enhancing mRNA vaccine efficacy against solid tumor models.

## 2 Materials and methods

### 2.1 Acquisition and processing of TCGA data

Bulk RNA-seq data and matched clinical information were obtained from TCGA cohorts through the UCSC Xena browser: LIHC (n = 423) and SKCM (n = 474). For survival analysis, only tumor samples were retained. Adjacent normal tissues were excluded, and when a patient had more than one tumor specimen, only the primary tumor was kept. Expression values were normalized to transcripts per million (TPM). Overall survival was measured from the date of surgical resection to death or last follow-up. Patients with zero survival time or missing vital status were removed. A cDC1 signature score was computed for each sample as the mean z-score of log_2_(TPM + 1) values across five genes: CLEC9A, XCR1, CLNK, THBD, and CADM1. cDC1 score was split at the cohort median into high and low groups for Kaplan-Meier curves and log-rank tests.

### 2.2 Acquisition and processing of single-cell RNA sequencing data

The scRNA-seq datasets analyzed in this study were obtained from the public Gene Expression Omnibus (GEO) database (https://www.ncbi.nlm.nih.gov/geo/). We collected HCC-related samples from GSE166635, GSE282701, and GSE242889 and melanoma-related samples from GSE221390 and GSE277165. The samples were classified into the tumor group and the normal group based on their source (13 tumors and 11 adjacent normal tissues for HCC; 12 tumors and 3 nevi for melanoma) and all included datasets were generated from whole tissue dissociation without CD45^+^ enrichment or FACS sorting.

Data processing was carried out using the Seurat package (v5.2.1) (25). Quality control was performed on the single-cell data, with RNA features ranging from 300 to 6000, RNA counts > 500, HB% < 1%, and MT% < 25%. The DoubletFinder package (v2.0.5) (26) was used to remove doublets. Further data integration was conducted using the Harmony package (v1.2.3) (27) to eliminate batch effects, followed by Uniform Manifold Approximation and Projection (UMAP) dimensionality reduction and clustering analysis. Finally, classic markers were used to annotate the cell clusters (**Supplementary Table 1**).

### 2.3 Analysis of myeloid cells and T/NK cell subpopulations

The annotated cell subpopulations were extracted using the subset function, and normalization, Harmony package (v1.2.3) removal of batch effects, and dimensionality reduction clustering analysis were performed again. Cell subpopulations were annotated using myeloid cell and T/NK cell markers (**Supplementary Table 2**), and the percentage of subpopulation cells was calculated. XCR1 expression specificity was quantified as the percentage of XCR1^+^ cells within each immune subset. cDC1 antigen-presentation (HLA-DRA, HLA-DRB1, CD74, CIITA, B2M, TAP1, TAP2, PSMB8, PSMB9) and maturation (CD40, CD80, CD83, CD86, LAMP3, CCR7, IL12B, CXCL9, CXCL10) programs were scored using AddModuleScore, and module scores were aggregated per sample for patient-level comparisons. MDSC-like populations were profiled with curated PMN-MDSC (S100A8, S100A9, S100A12, ARG1, OLFM4, CEACAM8, LCN2, MMP8, MMP9, DEFA4) and M-MDSC (FCN1, VCAN, CD14, S100A9, ARG1, PTGS2, IL1B, CXCL8, NAMPT, CHI3L1) gene sets.

### 2.4 Cell communication analysis

This study used the R package CellChat (v2.1.2) (28) to conduct a systematic analysis of intercellular interactions in single-cell transcriptome data. The annotated myeloid cells and T/NK cells were extracted, and they were classified into the tumor and normal groups based on the sample source. The analysis referred to the CellChatDB. Human database, focusing on Secreted Signaling and Cell-Cell Contact signaling pathways. In the data preprocessing stage, the identifyOverExpressedGenes and identifyOverExpressedInteractions functions were used to identify significantly expressed ligands and receptors in each cell population.

### 2.5 mRNA design and synthesis

The mRNA sequences encoding human GPC3, mouse XCL1 (mXCL1), mouse CD40L (mCD40L), the mouse XCL1-CD40L fusion protein (mXCL1-mCD40L), and the HPV-associated H658 antigen were constructed. The H658 mRNA vaccine encoded E6/E7 antigens derived from HPV16, HPV18, and HPV52. In the fusion construct, full-length coding sequences of mXCL1 and mCD40L were connected in frame using a (G4S)_3_ linker. The 5′ and 3′ UTRs were derived from human β-globin (HBB), and the poly(A) tail was encoded by the plasmid template as 100–120 consecutive adenines. The complete nucleotide and amino-acid designs are provided in **Supplementary Figure 1**.

The plasmids containing mRNA encoding sequences were linearized using BspQI (Vazyme) at 50 °C for 60 minutes. Then, an *in vitro* transcription reaction was performed using a co-transcription kit (DD4203, Vazyme) at 37 °C for 2 hours. Co-transcriptional capping with the CAG trimer cap analog produced mRNA with a native Cap 1 structure, and uridine was completely substituted with N1-methylpseudouridine triphosphate (N1-Me-Pseudo UTP). After the reaction, DNase I was added at 37 °C for 15 minutes to remove residual plasmid templates. Then, RNA was purified using magnetic beads (N412, Vazyme) without extra dsRNA removal, and the integrity of the purified mRNAs was confirmed by agarose gel electrophoresis (**Supplementary Figure 2**).

### 2.6 Prediction of mRNA-encoded protein structure

We used AlphaFold 3 (29) to model and analyze the tertiary structures of mXCL1, mCD40L, and their fusion proteins, verifying the structural integrity and spatial positions of each functional domain (chemokine folding conformation and TNF superfamily trimer structure) after fusion.

### 2.7 Preparation of mRNA-LNP and lyophilized formulations

SM102, DSPC, Cholesterol, and PEG-2000 were dissolved in anhydrous ethanol at a molar ratio of 50:10:38.5:1.5 (30). The purified mRNA was diluted in 50 mM sodium acetate buffer (pH 4.0) to a final concentration of 0.13 mg/mL. Then, the lipid and mRNA were mixed at a flow rate ratio of 1:3 using a microfluidic device. The particle size and polydispersity index (PDI) were determined using the Stunner platform (Unchained Labs). The encapsulation efficiency (EE, %) was determined using the Quant-iT RiboGreen RNA Assay Kit (R11490, Thermo Fisher) (31).

The encapsulated mRNA-LNP solution was diluted to 0.4 mg/mL, and the lyophilization protective agent (final concentration of 7.5% sucrose and 7.5% trehalose, w/v) was added before lyophilization. The lyophilization procedure: -50 °C, pre-freezing for 5 hours. Then, control vacuum at 3–5 Pa, one-time drying at -35 °C gradient heating to -15 °C, 45 hours, and secondary drying at 15 °C, 15 hours.

LNP particle size, PDI, and encapsulation efficiency for all formulations used in this study are provided in **Supplementary Table 3**.

### 2.8 Cell culture and transfection

HEK-293T, B16F10, TC-1 and RAW264.7 cell lines were purchased from Hunan Fenghui Biotechnology Co., Ltd. HEK-293T, B16F10 and RAW264.7 cells were cultured in DMEM (11965092, Gibco) supplemented with 10% fetal bovine serum (FBS) and 1% penicillin-streptomycin. TC-1 cells were cultured in RPMI 1640 (11875093, Gibco) supplemented with 10% FBS and 1% penicillin-streptomycin.

To establish stable cell lines, B16F10 cells were transduced with lentiviral vectors encoding human glypican-3 (hGPC3) and E6/E7 of HPV16/18/52, respectively. Forty-eight hours after transduction, cells were subjected to antibiotic selection using puromycin (2 μg/mL) for 14 days to obtain a stable cell population. RAW264.7 cells were transfected with 1 μg of luciferase (Luc) mRNA-LNP as the control group and equal amounts of mXCL1-mCD40L mRNA-LNP for the experimental group, respectively. After 24 hours, cells were collected and RNA was extracted for RT-qPCR experiments.

Mouse peritoneal macrophages: Take 6–8 weeks old C57BL/6J mice, and inject 1 ml of 3% thioglycollate broth intraperitoneally once a day. After 3 days, sacrifice the mice, fix them in a supine position, inject 5 ml of pre-cooled DPBS into the peritoneum, and gently massage the abdomen for 3–5 minutes. Use a sterile syringe to repeatedly rinse the peritoneal cavity twice, and collect the washings. Place the mouse washings at room temperature and centrifuge at 1000 rpm for 10 minutes, then resuspend the cells in RPMI 1640 (containing 10% fetal bovine serum and 1% penicillin-streptomycin) and place them in a 6-well plate for static culture. After 2–3 h of adherence culture, non-adherent cells were removed, fresh RPMI 1640 complete medium was added, and cells were treated with 1 μg of Luciferase (Luc) mRNA-LNP or 1 μg of mXCL1-mCD40L mRNA-LNP. After 24 hours, collect the cells and extract RNA for RT-qPCR experiments.

Mouse BMDC cells: Take 6–8 weeks old C57BL/6J mice, separate their femurs and tibias. Wash with DPBS buffer, use gauze to peel and clean the attached muscle tissue on the bone surface. Rinse the bone again with DPBS, cut off the ends of the bone, and use a syringe to aspirate the bone marrow cavity. Collect the washed bone marrow cell suspension into a 50 ml centrifuge tube. Centrifuge at 1000 rpm for 5 minutes, discard the supernatant. Add 4 ml of ACK lysis solution to resuspend the cell precipitate to remove red blood cells. Centrifuge again at 1000 rpm for 5 minutes. Discard the supernatant, and resuspend the cells in 10 ml of RPMI1640 complete culture medium and inoculate into Petri dishes. The culture medium should contain 20 ng/ml GM-CSF and 10 ng/ml IL-4. On the 3rd day, add 10 ml of DC culture medium (total 20 ml) to each dish. On the 6th day, aspirate 10 ml of the culture supernatant and centrifuge, discard the supernatant, and resuspend the cell precipitate in an equal volume of fresh DC culture medium, then re-add it to the original dish. On day 8, add Luc mRNA-LNP (control group) and mXCL1-mCD40L mRNA-LNP (experimental group) each at 1 μg. After 24 hours, collect the cells and extract RNA for RT-qPCR experiments.

### 2.9 RNA extraction and reverse transcription-quantitative PCR analysis

Transfected cells or tissues were harvested, and total RNA was extracted using the RNA Easy Fast Animal Tissue/Cell Total RNA Kit (DP451, Tiangen). Reverse transcription was performed using the HiScript III 1st Strand cDNA Synthesis Kit (+gDNA wiper) (R31302, Vazyme).

qPCR was performed using ChamQ Universal SYBR qPCR Master Mix (Q711-03, Vazyme), where *Gapdh* was used as the internal reference for normalization. Relative expression levels are calculated using the 2^(-ΔΔCt) method. The sequences of the involved genes and primers are shown in **Supplementary Table 4**.

### 2.10 Animal works

Female C57BL/6 mice (specific pathogen-free, 6–8 weeks old, 18–22 g) were purchased from Beijing SPF Biotechnology Co., Ltd. and randomly divided into experimental groups. All animal experiments were conducted in accordance with institutional guidelines and approved by the Animal Welfare and Ethics Committee of Beijing Institute of Biological Products Co., Ltd. (Approval No.: BSYYF20250813003). Mice were anesthetized by inhalation of 4% isoflurane before procedures such as drug administration and tumor measurement. At the experimental endpoint, mice were euthanized by CO_2_ inhalation at a flow rate displacing 30%–70% of the chamber volume per minute; death was confirmed by cessation of breathing and heartbeat, followed by cervical dislocation as a secondary method to ensure death. Animals were randomly allocated to treatment groups using stratified randomization based on initial tumor volume. Tumor volumes were measured by the same trained investigator throughout the study to ensure consistency. However, cages were labeled with group identifiers, so the investigator was not formally blinded to group allocation. Sample sizes (n = 5–9 per group depending on the different experiments) were based on previously published tumor efficacy studies of similar design and on the 3R principles to limit animal use without a formal power analysis. Mice that failed to establish measurable tumors after inoculation were excluded from analysis.

#### 2.10.1 Therapeutic subcutaneous B16F10-hGPC3 tumor-bearing mouse model

Due to the spontaneous regression of Hepa1–6 tumors observed in immunocompetent C57BL/6 mice during pilot studies, the B16F10 cell line was selected for its robust, consistent tumorigenicity. Luc mRNA served as the dose-matched irrelevant control, with matching Cap1 capping, N1-Me-Pseudo U modification, purification workflow and similar transcript length relative to GPC3 mRNA. To establish the mouse subcutaneous tumor-bearing treatment model, B16F10-hGPC3 cells (5 × 10^5^ cells per mouse) were subcutaneously injected into the right flank of each mouse on Day 0 (D0). On Day 7 (D7), when the tumors became palpable, the mice were randomly divided into 5 groups (n = 9 per group) and received the following treatments via intramuscular (i.m.) injection: Control group: Luc mRNA-LNP (12 μg per dose). Experimental group: GPC3+Luc mRNA-LNP (9 μg + 3 μg per dose); GPC3+mXCL1 mRNA-LNP (9 μg + 3 μg per dose); GPC3+mCD40L mRNA-LNP (9 μg + 3 μg per dose); GPC3+mXCL1-mCD40L mRNA-LNP (9 μg + 3 μg per dose). The treatments were administered on D7, D11, D15, and D20. When the tumor volume reached 2000 mm^3^ or on D35, the mice were sacrificed.

#### 2.10.2 Therapeutic efficacy of GPC3 mRNA vaccine combined with fusion or cocktail adjuvant mRNAs

B16F10-hGPC3 cells (5 × 10^5^ cells per mouse) were subcutaneously injected into the right flank of C57BL/6 mice on Day 0. On Day 6, when the tumors became palpable, the mice were randomly divided into five groups (n = 9 per group) and received intramuscular (i.m.) injections on D6, D9, D12, and D15 as follows: control group, DPBS and Luc mRNA-LNP (12 μg per dose); GPC3 +Luc mRNA LNP (9 μg + 3 μg per dose); GPC3+mXCL1+mCD40L mRNA-LNP (9 μg + 1.5 μg + 1.5 μg per dose); and fusion group, GPC3+mXCL1-mCD40L mRNA-LNP (9 μg + 3 μg per dose). All groups received a matched total mRNA dose of 12 μg per injection. Mice were euthanized when tumor volume reached 2000 mm³ or on Day 24, at which point spleens were harvested for IFN-γ ELISPOT analysis. To confirm stable hGPC3 expression, total RNA was extracted from B16F10-hGPC3 tumors collected before and after treatment and from B16F10-WT tumors, and hGPC3 mRNA was quantified by qRT-PCR.

#### 2.10.3 Mouse model for GFP-labeled tumor antigen uptake by cDC1

To establish an *in vivo* mouse model for assessing tumor antigen uptake by DCs, B16F10-GFP and B16F10-WT cells (5 × 10^5^ cells per mouse) were subcutaneously injected into the right flank of each mouse on Day 0 (D0). On Day 5 (D5), when the tumors became palpable, The experimental setup comprised four groups: Luc mRNA-LNP (3 μg), mXCL1+mCD40L mRNA-LNP (1.5 μg + 1.5 μg per dose), mXCL1-mCD40L mRNA-LNP (3 μg) and mXCL1-mCD40L mRNA-LNP in B16F10-WT (3 μg) (n = 5 per group). Muscle injections were administered on D5, D7, and D9, and draining lymph nodes (dLNs) were collected from the mice on D11 for subsequent analyses (32).

#### 2.10.4 B16-OVA cross-presentation assay

The B16-OVA cell line and corresponding plasmids were kindly provided by Professor Yangxin Fu, Tsinghua University. B16F10-OVA cells (5 × 10^5^ cells per mouse) were subcutaneously injected into the right flank of C57BL/6 mice on Day 0. On Day 6, when the tumors became palpable, the mice were randomly divided into four groups (n = 5 per group) and received intramuscular injections on D6, D9 and D12 as follows: control group, Luc mRNA-LNP (12 μg per dose); OVA+Luc mRNA-LNP (9 μg + 3 μg per dose); OVA+mXCL1+mCD40L mRNA-LNP (9 μg + 1.5 μg + 1.5 μg per dose); and fusion group, OVA+mXCL1-mCD40L mRNA-LNP (9 μg + 3 μg per dose). All groups received a matched total mRNA dose of 12 μg per injection. dLNs and tumors were harvested on Day 14 for flow cytometric analysis.

#### 2.10.5 Safety assessment mouse model for GPC3 mRNA vaccine combined with mXCL1-mCD40L adjuvant

To establish the GPC3 combined with adjuvant safety and efficacy model, direct muscle injections were administered on D1, D5, and D9, and peripheral blood of the mice was collected 4 hours, 24 hours after D1 administration, and on D11 for subsequent detection. The mice were divided into 4 groups: DPBS, Luc (12 μg), GPC3+Luc (9 μg + 3 μg), and GPC3+mXCL1-mCD40L (9 μg + 3 μg) (n = 6 per group). Serum was collected at 4 hours and 24 hours after the first administration to detect liver function indicators ALT and AST.

#### 2.10.6 Therapeutic subcutaneous B16F10-H658 tumor-bearing mouse model

To establish early therapeutic mouse models, B16F10-H658 cells, defined as a 1:1:1 mixture of B16F10 cells stably expressing HPV16 E6/E7, HPV18 E6/E7, or HPV52 E6/E7, were subcutaneously injected into the right flank of C57BL/6 mice at 4.5 × 10^5^ cells per mouse on Day 0 (D0). TC-1 cells were subcutaneously injected into the right flank of C57BL/6 mice at 5 × 10^5^ cells per mouse on Day 0 (D0). On Day 3 (D3), the mice were randomly divided into 3 groups (n = 9 per group) and received the following treatments via intramuscular injection: Control group: DPBS. Experimental group: H658+Luc mRNA-LNP (15 μg + 3 μg per dose) and H658+ mXCL1-mCD40L mRNA-LNP (15 μg + 3 μg per dose). The treatments were administered on D3, D6, D9, and D12.

#### 2.10.7 Regional DC maturation in ipsilateral versus contralateral dLNs

Regional DC maturation in ipsilateral versus contralateral dLNs. B16F10 cells (5 × 10^5^ per mouse) were subcutaneously injected into the right flank of C57BL/6 mice on Day 0. On Day 5, mice were randomly divided into three groups (n = 5 per group) and received intramuscular injections of DPBS, 3 μg Luc mRNA-LNP, or 3 μg mXCL1-mCD40L mRNA-LNP on Days 5, 7, and 9. On Day 11, ipsilateral and contralateral dLNs were harvested separately, and mature DCs (CD11c^+^MHC-II^+^CD86^+^) were quantified by flow cytometry.

#### 2.10.8 Comparison of intratumoral and intramuscular administration routes

Comparison of intratumoral and intramuscular administration routes. B16F10-H658 tumor-bearing mice were established as described above. Luc (18 μg), Luc+mXCL1 (15 μg + 3 μg), Luc+ mCD40L (15 μg + 3 μg), Luc+mXCL1-mCD40L (15 μg + 3 μg) and H658+mXCL1-mCD40L mRNA-LNP (15 μg + 3 μg) were administered either intratumorally or intramuscularly on D7, D10, D13 and D16 (n = 9 per group), with Luc mRNA-LNP as the route-matched control. Tumor growth was monitored as above.

### 2.11 Biodistribution of intramuscularly injected mRNA-LNP

Biodistribution of intramuscularly injected mRNA-LNP. For bioluminescence imaging, mice received intramuscular injection of 25 μg Luc mRNA-LNP; D-luciferin (150 mg/kg) was administered intraperitoneally 4 h later, and images were acquired 10–15 min thereafter. For cellular-level biodistribution, mice bearing subcutaneous B16F10 tumors received intramuscular injection of 20 μg mXCL1-mCD40L-GFP mRNA-LNP. Sixteen hours later, the injection-site muscle, bilateral dLNs, spleen, kidney, part of the liver, and tumor were harvested, and GFP^+^ cells in each tissue were quantified by flow cytometry.

### 2.12 Flow cytometry analysis

For tissue-derived cells, tissues were ground and filtered to generate a single-cell suspension; for peripheral blood samples, red blood cells were subjected to lysis using ACK lysis buffer. All single-cell suspensions were subsequently washed and collected with phosphate-buffered saline (PBS). Fc receptors were blocked with anti-CD16/32 (156604) antibodies to eliminate non-specific binding. The cell suspension was centrifuged at 1000 rpm for 5 min at 4 °C, and the supernatant was carefully discarded. Cell pellets were resuspended in flow cytometry staining buffer for subsequent immunofluorescence staining. For immunofluorescence staining, the cell suspension was incubated with 0.5 μL of anti-CD11c (117349), 0.5 μL of anti-CD103 (156905), 0.5 μL of anti-MHC II (107605), 0.5 μL of anti-CD86 (105007), 0.5 μL of anti-CD45 antibody (103130), 0.5 μL of anti-CD3 (100306), 0.5 μL of anti-CD8a (100724), 0.5 μL of anti-mouse H-2K^b^ bound to SIINFEKL (141603) and 0.5 μL of Zombie NIR Fixable Viability Dye (B427488) at 4°C for 20 min in the dark. CD11c^+^CD103^+^ cells were analyzed as a cDC1-enriched DC population. After incubation, the stained cells were centrifuged at 1000 rpm for 5 min at 4°C, and the supernatant was discarded. Cell pellets were resuspended in fresh flow cytometry staining buffer, then filtered through a 70 μm cell strainer to remove cell clumps. Flow cytometric analysis was performed using a CytoFlex S flow cytometer. All flow cytometry gating strategies are shown in **Supplementary Figure 3**.

### 2.13 Liver toxicity assessment

Alanine aminotransferase (ALT) and aspartate aminotransferase (AST) activities were measured using an ALT Assay Kit (C009-2-1) and an AST Assay Kit (C010-2-1), respectively. Briefly, 20 μL pre-warmed reagent was mixed with 5 μL sample in 96-well plates and incubated at 37 °C for 30 min. Then, 20 μL chromogenic reagent (2,4-dinitrophenylhydrazine for ALT, Reagent II for AST) was added, followed by incubation at 37 °C for 20 min. After the addition of 200 μL 0.4 mol/L NaOH and 15 min incubation at room temperature, absorbance was detected at 505 nm (ALT) or 510 nm (AST) using a microplate reader.

### 2.14 Enzyme-linked immunosorbent assay

HEK-293T cells were seeded in 6-well plates and treated with mXCL1-mCD40L and control Luc mRNA-LNP. At 24 h after treatment, cell supernatant and lysates were collected. The expression of the encoded fusion protein was evaluated using ELISA kits specific for mouse XCL1 (RD-RX21901, Henghuibio), CD40L (SEKM-0296, Solarbio) according to the manufacturer’s instructions.

Mouse IL-12p70 levels were detected using an ELISA Kit (SEKM-013, Solarbio) following the manufacturer’s protocol. Briefly, ELISA plates were washed twice with wash buffer. Standards and samples were added and incubated at 37 °C for 90 min. After washing, biotinylated detection antibody and enzyme conjugate were added sequentially with appropriate incubations. TMB substrate was then added for color development at room temperature. The reaction was stopped, and absorbance was measured at 450 nm using a Varioskan LUX microplate reader (Thermo Fisher Scientific).

### 2.15 Transwell migration assay

To assess the chemotactic activity of mXCL1-mCD40L toward dendritic cells, B16F10 cells (1 × 10^5^ per well) were seeded into the lower chamber of a 24-well transwell plate. On the following day, the cells were separately transfected with mXCL1-mCD40L, mXCL1, or control Luc mRNA-LNP. When the cells reached 70–80% confluence, the culture medium was replaced with serum-free RPMI 1640. BMDCs, generated as described above, were resuspended in serum-free RPMI 1640, and 5 × 10^4^ cells were added to the upper chamber of a transwell insert with a 5 μm pore size. After incubation at 37 °C in 5% CO_2_ for 12 h, the cells in the lower chamber were collected and analyzed by flow cytometry (10000 events acquired per sample). Migrated BMDCs were identified as CD11c^+^MHC-II^+^ cells and quantified.

### 2.16 IFN-γ ELISpot assay

Single-cell suspensions were prepared by mechanical dissociation through a 70 μm cell strainer, followed by red blood cell lysis with ACK lysis buffer. After washing, splenocytes were resuspended in complete RPMI 1640, and viable cells were counted.

IFN-γ-producing antigen-specific T cells were detected using a mouse IFN-γ ELISpot kit (ESP-M0001S, Elabscience). Splenocytes were seeded at 2 × 10^5^ cells per well in a pre-coated 96-well ELISpot plate and stimulated with a pool of five hGPC3 peptides (2 μg/mL, each peptide). Wells incubated with medium alone served as negative controls. For positive controls, 5 × 10^4^ splenocytes per well were stimulated with PMA (50 ng/mL) plus ionomycin (1 μg/mL). After incubation at 37 °C in 5% CO_2_ for 24 h, the plates were developed according to the manufacturer’s instructions, and spots were enumerated using an automated ELISpot reader. The number of GPC3-specific IFN-γ spot-forming cells was calculated by subtracting the mean spot count of the medium-alone wells from that of the peptide-stimulated wells, and results are expressed as IFN-γ spots per 10^6^ splenocytes.

### 2.17 Statistical analysis

The statistical analysis methods for single-cell transcriptome data are detailed in the previous relevant sections. *In vitro* experiments were conducted with at least three independent replicates, and data were expressed as mean ± SEM. Two-group comparisons were performed with two-tailed unpaired t-tests, and multiple groups were compared by one-way ANOVA with Tukey’s post test, unless otherwise indicated. Subsequent *in vitro* and *in vivo* experimental data were analyzed using R software. Tumor growth curves were analyzed by linear mixed-effects models. The tumor growth rate of each group was estimated as the model-derived slope (mm³/day). Pre-specified pairwise comparisons of growth rates among the treatment groups were performed, and P values were adjusted for multiple testing using the Holm–Bonferroni method. Areas under the curve (AUC) were compared by one-way ANOVA. Individual tumor growth curves are shown for all efficacy experiments. Mice were euthanized when tumor volume reached 2000 mm³, and this was recorded as a death event in the survival analysis. Mice alive at the end of the experiment were censored. Kaplan-Meier survival curves were compared using the log-rank test. (*P < 0.05, **P < 0.01, ***P < 0.001, ****P < 0.0001).

## 3 Results

### 3.1 Prognostic value of a cDC1 signature and single-cell landscape of the tumor microenvironment in HCC and melanoma

Using bulk RNA-seq data from the TCGA-LIHC and TCGA-SKCM cohorts (**Figure 1A**), we calculated the cDC1 signature score for each tumor sample based on the expression of five core cDC1 genes: CLEC9A, XCR1, CLNK, THBD, and CADM1. Furthermore, we dichotomized patients according to the cohort-specific median of cDC1 signature score. We found high cDC1 scores were associated with longer overall survival in both cohorts (LIHC, log-rank *P* = 0.01; SKCM, *P* = 4.9 × 10^-4^, **Figure 1B**).

**Figure 1:**
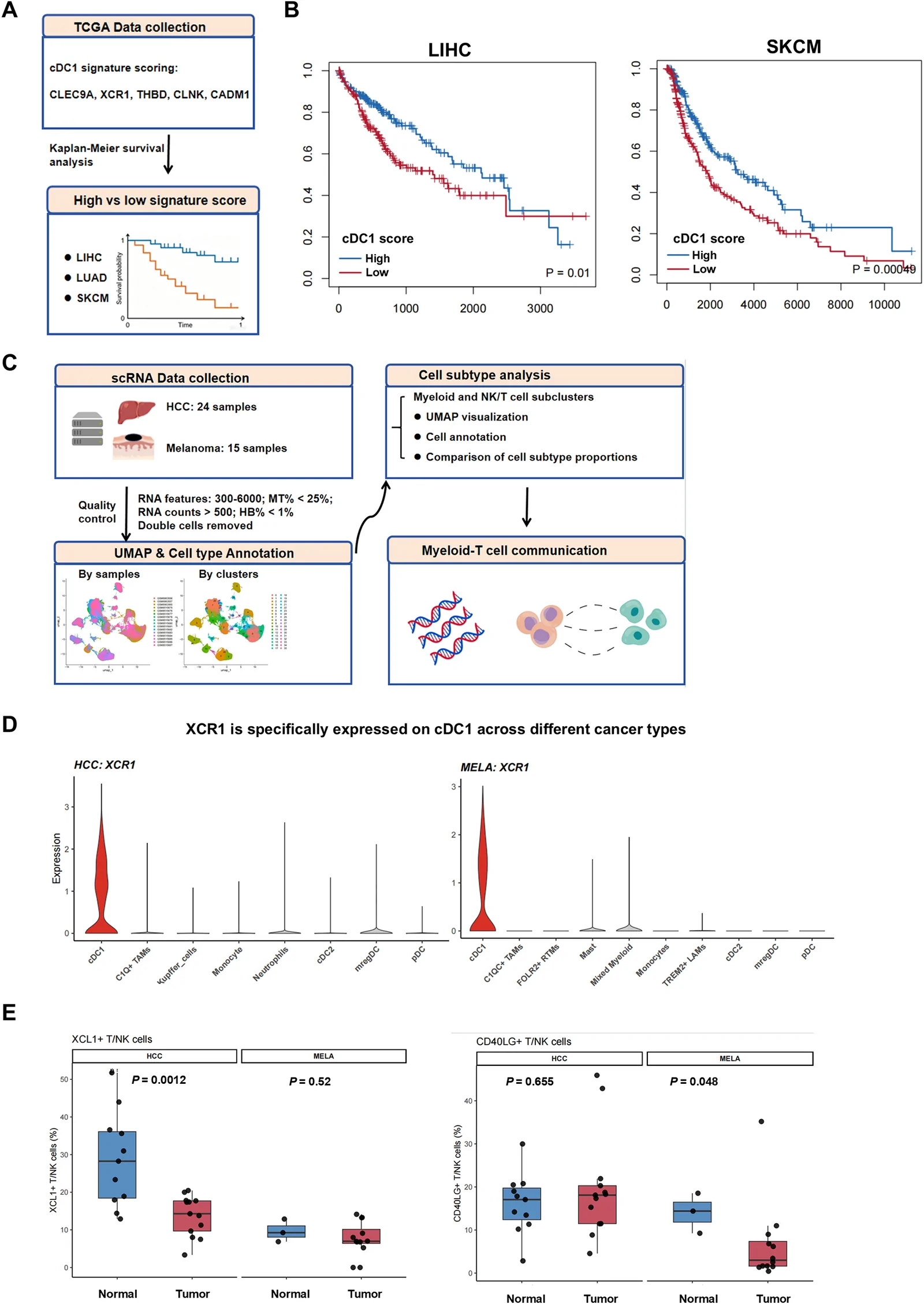
Prognostic value of the cDC1 signature and single-cell characterization of cDC1-related immune axes in human HCC and melanoma. **(A)** Schematic overview of the analytical workflow for TCGA bulk RNA-seq data, including cDC1 signature scoring and Kaplan-Meier survival analysis. **(B)** Kaplan-Meier overall survival curves of patients with high versus low cDC1 signature scores in the LIHC (left) and SKCM (right) cohorts from TCGA. **(C)** Schematic overview of the study workflow for pan-cancer single-cell RNA sequencing scRNA-seq analysis. **(D)** Violin plots showing the expression profile of XCR1 across different myeloid cell subsets in HCC (left) and melanoma (right). **(E)** Box-and-whisker plots comparing the proportions of XCL1+ and CD40LG+ T/NK cells between tumor and adjacent normal tissues in HCC and melanoma cohorts.

We then collected sample data of different cancer types from the GEO database and performed separate integrated analyses (**Figure 1C**), including scRNA-seq data from 24 HCC and 15 melanoma samples (**Supplementary Figure 4**). Based on the expression patterns of well-established cell-type markers, all cells were annotated as the following main cell types (**Supplementary Figure 5A**).

Further myeloid cell clustering analysis (**Supplementary Figures 5B, C**) revealed cDC1 proportions of 5.5% in melanoma and 4.4% in HCC within the myeloid compartment. Notably, cDC1 frequencies showed no significant difference between tumor and normal samples across cancer types (**Supplementary Figures 6A, B**).

At the patient level, antigen presentation and maturation module scores were similar between tumor and normal cDC1s in both cancer types (all *P* > 0.1; **Supplementary Figure 7**). We next performed cell-cell communication analysis between myeloid cells and T/NK cells using CellChat (**Figure 1E**). Immune-activating pathways were markedly reduced in tumors. XCL1-XCR1 signaling was significantly reduced in HCC, whereas CD40-CD40L signaling was reduced in melanoma, indicating that each tumor type disrupts a distinct arm of DC-T cell costimulation and licensing. Thus, the XCL1-XCR1 and CD40-CD40L signaling axis may serve as promising therapeutic targets.

To test if myeloid-derived suppression drives cDC1 dysfunction, we scored PMN-MDSC and M-MDSC signatures across myeloid subsets (**Supplementary Figure 8A**). No expansion of MDSC-like programs in tumor versus normal tissues (**Supplementary Figure 8B**) rules out suppressive myeloid cell expansion as the cause, highlighting the need for exogenous maturation signals like CD40 ligation to restore cDC1 function in the TME.

Meanwhile, XCR1 was significantly enriched in cDC1 (**Figure 1D**). To quantify the targeting specificity underlying our ligand choice, we measured XCR1 expression across all immune subsets. XCR1 was selectively expressed by cDC1s (58.5% and 48.3% XCR1^+^ cells among cDC1s in HCC and melanoma), whereas fewer than 5% of cells in all other myeloid and lymphoid subsets were XCR1^+^ (**Supplementary Figure 9**). This restricted expression pattern supports XCL1 as the cDC1-targeting domain of the fusion construct.

Collectively, cDC1s in these tumors receive diminished input from activating ligands, particularly via XCL1 and CD40-mediated signaling axes. Given the critical role of cDC1s in anti-tumor immunity, we generated an mXCL1-mCD40L fusion mRNA to promote cDC1 recruitment and maturation and thereby improved anti-tumor efficacy.

### 3.2 Design and *in vitro* characterization of the dual-function mXCL1-mCD40L mRNA adjuvant

Based on the above bioinformatics analyses, we designed a dual-functional mRNA adjuvant to simultaneously enhance cDC1-directed chemotaxis and DC maturation. We individually constructed mRNA molecules encoding mouse XCL1 (mXCL1), CD40L (mCD40L), and the XCL1-CD40L fusion mRNA (mXCL1-mCD40L). In the fusion construct, XCL1 and CD40L were connected by the (G_4_S)_3_ linker. All designed molecules contained a 5’ UTR, Kozak sequence, 3’ UTR, and poly(A) tail to ensure efficient translation and stability of the mRNA (**Figure 2A**).

**Figure 2:**
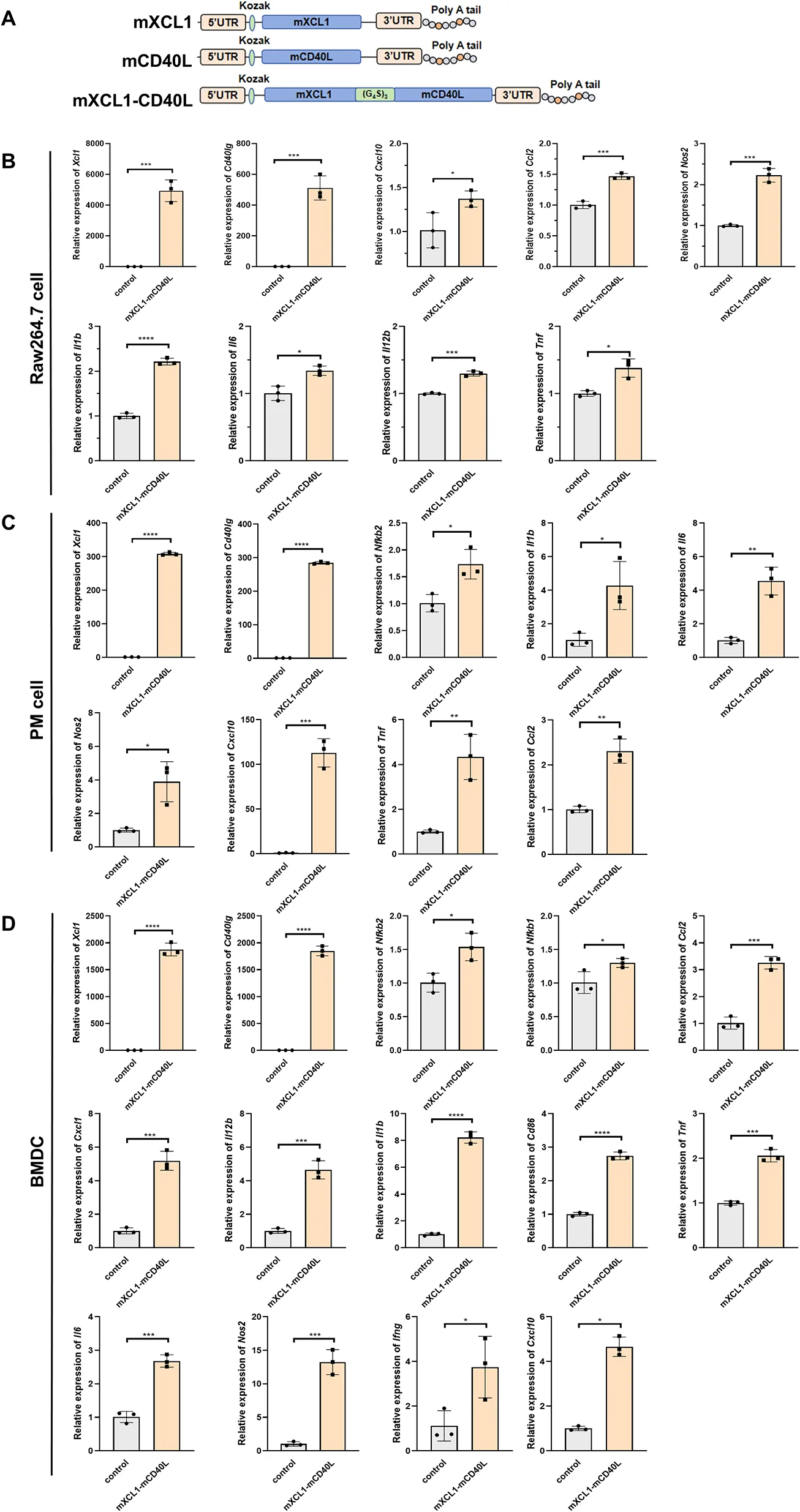
Design of the mXCL1-mCD40L fusion mRNA and its immune activation activity in myeloid cells *in vitro*. **(A)** Schematic diagram of mXCL1-mCD40L mRNA structure. The fusion construct contains mXCL1 and mCD40L domains connected by a GS linker, with 5’ UTR, Kozak sequence, and 3’ UTR with poly**(A)** tail. **(B)** RAW264.7 cells were treated with control or mXCL1-mCD40L, followed by measurement of immune-related gene expression by qRT-PCR. **(C)** PMs were treated with control or mXCL1-mCD40L, followed by measurement of immune-related gene expression by qRT-PCR. **(D)** BMDCs were treated with control or mXCL1-mCD40L, followed by measurement of immune-related gene expression by qRT-PCR. Data are presented as mean ± SEM. *P < 0.05, **P < 0.01, ***P < 0.001, ****P < 0.0001.

To characterize the immunostimulatory properties of the mXCL1-mCD40L mRNA-LNP, we performed a series of *in vitro* validation experiments. We first treated RAW264.7 cells with Luc mRNA-LNP or mXCL1-mCD40L mRNA-LNP and the expression of downstream immune-related genes was detected by qPCR (**Figure 2B**). The results demonstrated that the mXCL1-mCD40L group exhibited a significant upregulation effect in multiple immune activation indicators. Among inflammatory mediators, mXCL1-mCD40L induced the expression of *Nos2* (*P* < 0.0001), indicating that the fusion protein activated the nitric oxide synthase pathway and triggered downstream inflammatory response and immune activation program. The mRNA levels of pro-inflammatory cytokines *Il1b*, *Il12b*, *Il6*, and *Tnf* were significantly increased. The expression of chemokines *Cxcl10* and *Ccl2* was also significantly increased, suggesting that mXCL1-mCD40L mRNA-LNP promotes chemokine gene induction in macrophages.

We further explored the immune activation mechanism in primary immune cells. Mouse peritoneal macrophages and bone marrow-derived dendritic cells (BMDCs) were isolated and treated *in vitro* with control (Luc mRNA-LNP) or mXCL1-mCD40L mRNA-LNP formulations. We subsequently analyzed the expression changes of immune-related genes. In mouse peritoneal macrophages, mXCL1-mCD40L mRNA-LNP treatment significantly upregulated pro-inflammatory cytokines *Tnf*, *Il1b*, and *Il6*, as well as *Nos2* expression; the NF-κB pathway-related gene *Nfkb2* was also elevated. The expression of chemokines *Ccl2* and *Cxcl10* significantly increased, suggesting that mXCL1-mCD40L mRNA-LNP induces the production of Th1-type chemokines (**Figure 2C**). In BMDCs, mXCL1-mCD40L mRNA-LNP also exhibited an activating effect. The key pro-inflammatory factors *Il12b*, *Il1b*, *Il6*, and *Tnf* were all upregulated. The expression of NF-κB pathway-associated genes *Nfkb1* and *Nfkb2* increased, suggesting activation of NF-κB-related transcriptional programs in BMDCs. Notably, the mRNA level of DC maturation marker *Cd86* sharply elevated, *Nos2* expression was upregulated 13-fold, and the chemokines *Cxcl1*, *Ccl2*, and *Cxcl10* were all significantly elevated, and *Ifng* mRNA levels rose 4-fold. These results indicate that mXCL1-mCD40L mRNA-LNP promotes BMDC activation, with increased expression of inflammatory cytokines, chemokines, NF-κB-related genes, and the co-stimulatory molecule *Cd86* (**Figure 2D**). Collectively, these *in vitro* findings demonstrate that mXCL1-mCD40L mRNA-LNP robustly activates innate immune programs across multiple cell types, as evidenced by the coordinated upregulation of pro-inflammatory cytokines, Th1-polarizing chemokines, and co-stimulatory molecules.

### 3.3 Structural integrity and functional validation of the mXCL1-mCD40L fusion mRNA-LNP

We employed AlphaFold 3 to analyze the tertiary structures of mXCL1, mCD40L, and mXCL1-mCD40L, and to verify the structural integrity of the fusion protein. The results showed that mXCL1 adopted a typical chemokine folding conformation, while mCD40L maintained the characteristic domain of the TNF superfamily. Importantly, following fusion via the (G_4_S)_3_ flexible linker, the protein conformations of XCL1 and CD40L remained largely intact, indicating that the flexible linker effectively avoided spatial hindrance between the two functional domains, providing a structural basis for the fusion mRNA to simultaneously exert the dual functions of cDC1 chemotaxis and DC activation (**Figure 3A**). CD40L typically exists in its trimeric form to exert biological activity. Further structural prediction of the trimeric assembly of the TNF homology domain (THD) revealed that mXCL1-mCD40L maintained a trimeric THD assembly comparable to that of native mCD40L (**Figure 3B**).

**Figure 3:**
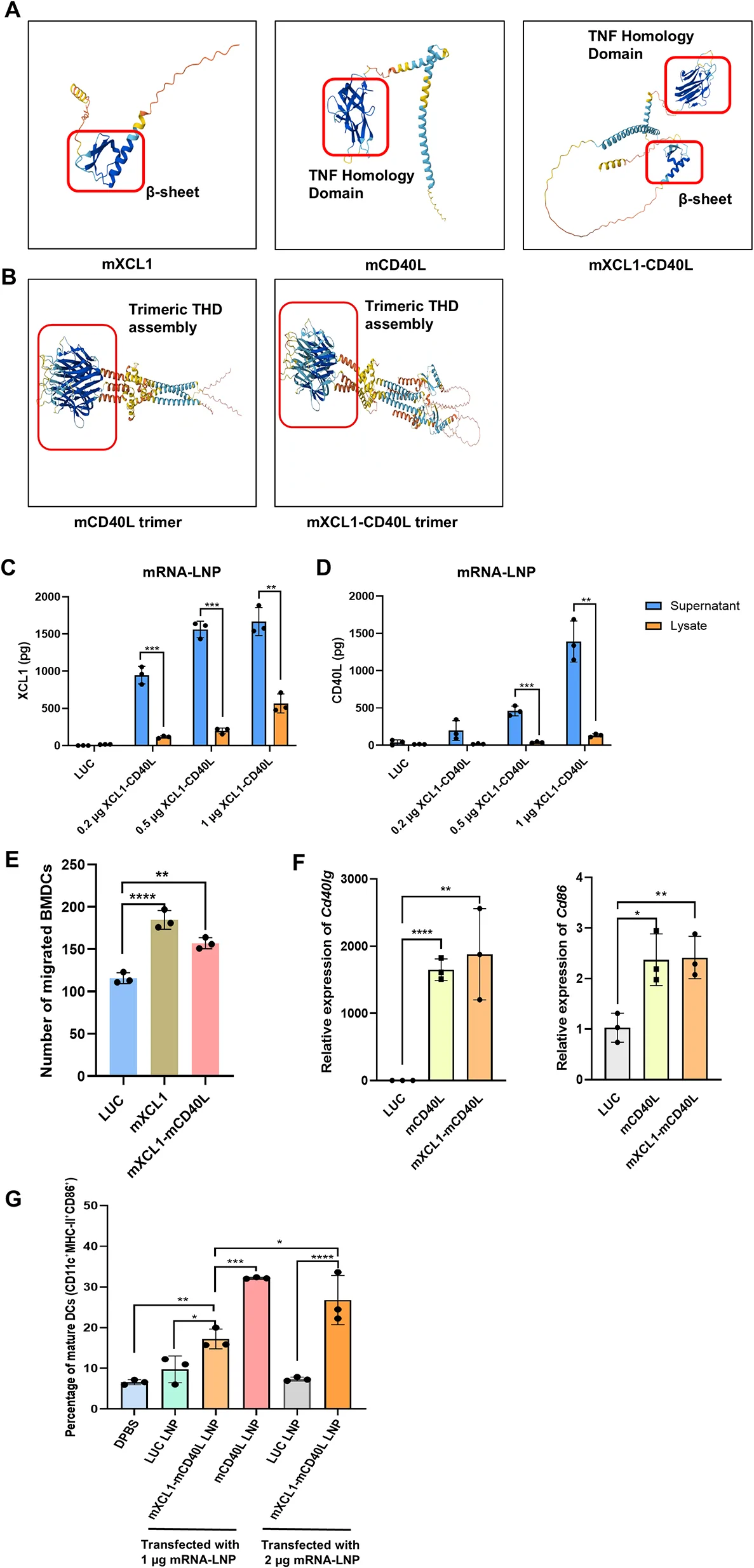
Structural integrity, expression characterization and functional validation of the mXCL1-mCD40L fusion protein. **(A)** Tertiary structure prediction of mXCL1, mCD40L, and mXCL1-mCD40L proteins based on AlphaFold 3. **(B)** Multimeric structure prediction of mCD40L trimer and mXCL1-mCD40L trimer showing trimeric THD assembly. **(C, D)** ELISA quantification of supernatant and lysate mXCL1 **(C)** and mCD40L **(D)** protein after transfection with increasing doses of mRNA-LNP (0.2 μg, 0.5 μg, 1 μg). **(E)** Transwell migration assay showing the number of migrated BMDCs induced by Luc, mXCL1, or mXCL1-mCD40L mRNA-LNP. **(F)** Relative mRNA expression of *Cd40lg* and *Cd86* in BMDCs treated with Luc, mCD40L, or mXCL1-mCD40L mRNA-LNP. **(G)** Flow cytometric quantification of mature BMDCs (CD11c^+^MHC-II^+^CD86^+^) after transfection with 1 μg or 2 μg of the indicated mRNA-LNP formulations. DPBS served as the control. Data are presented as mean ± SEM. *P < 0.05; **P < 0.01; ***P < 0.001; ****P < 0.0001.

To confirm that the engineered fusion mRNA could be translated into the expected protein product, cells were transfected with mXCL1-mCD40L mRNA-LNP, and the culture supernatant and cell lysates were collected separately for ELISA analysis. Both the XCL1 and CD40L proteins were detected at markedly higher levels in the culture supernatant than in the cell lysates. These findings validate the translational potential of the fusion mRNA construct and demonstrates that the fusion protein is efficiently secreted rather than retained on the plasma membrane. These suggested that both functional domains are released into the extracellular space, where they interact with their receptors XCR1 and CD40 (**Figures 3C, D**).

To validate the function of each domains of the fusion protein with XCL1 or CD40L, we performed transwell migration and DC maturation assays. In transwell migration assays, mXCL1 significantly promoted BMDC migration relative to the Luc control (*P* < 0.0001), and the mXCL1-mCD40L fusion mRNA likewise significantly increased BMDC migration (*P* < 0.01), although to a slightly lower extent than mXCL1 alone (**Figure 3E**). These results confirm that the XCL1 moiety retains chemotactic activity within the fusion protein. For DC maturation, qPCR analysis revealed that both mCD40L alone and mXCL1-mCD40L significantly upregulated the expression of *Cd40lg* and *Cd86* in BMDCs compared with the Luc control. Notably, mXCL1-mCD40L induced significantly higher *Cd40lg* expression than mCD40L alone, whereas the two treatments upregulated *Cd86* to a comparable extent (**Figure 3F**). We further verified DC maturation by flow cytometry and quantified mature BMDCs defined as CD11c^+^MHC-II^+^CD86^+^ cells. Consistent with the gene expression result, mXCL1-mCD40L mRNA-LNP significantly increased the percentage of mature BMDCs relative to both the DPBS and Luc mRNA-LNP controls (**Figure 3G**). The mXCL1-mCD40L fusion group exhibited a dose-dependent increase in the proportion of mature DCs across the 1 μg and 2 μg doses. The maturation efficacy of the 2 μg mXCL1-mCD40L dose was comparable to that of the 1 μg dose of CD40L alone. Collectively, these functional assays demonstrate that the mXCL1-mCD40L fusion protein still preserves XCR1-directed chemotaxis and CD40L mediated DC maturation, consistent with the structural predictions in **Figures 3A, B**.

### 3.4 The mXCL1-mCD40L mRNA-LNP enhances the anti-tumor efficacy of the GPC3 vaccine in tumor-bearing mouse models

To verify this synergistic therapeutic effect *in vivo*, we established a B16F10-hGPC3 subcutaneous tumor-bearing model in C57BL/6 mice, as illustrated in **Figure 4A**. Quantitative RT-PCR confirmed that B16F10-hGPC3 tumors stably expressed hGPC3 before and after treatment (**Supplementary Figures 10A** Left).

**Figure 4:**
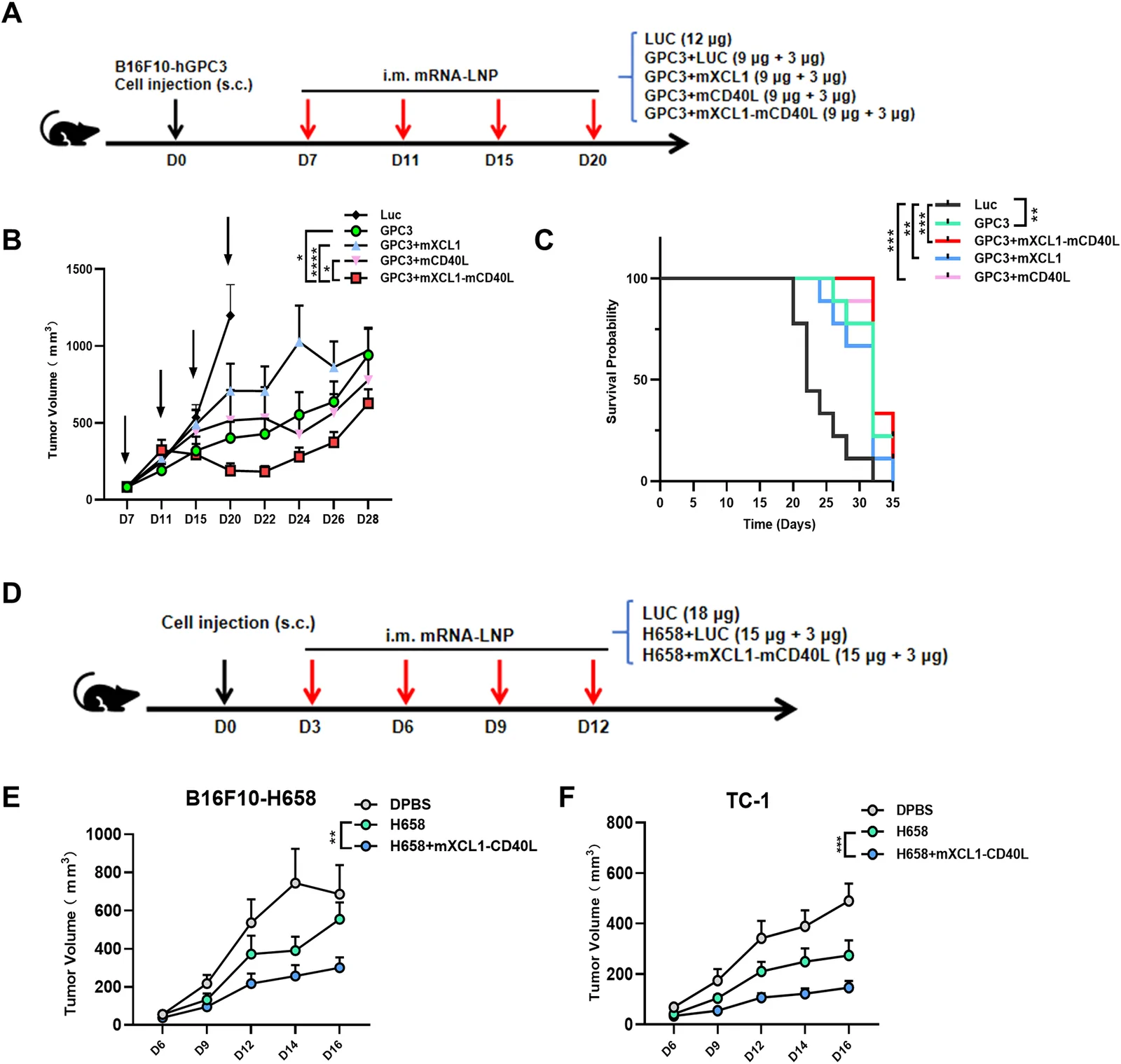
The mXCL1-mCD40L mRNA adjuvant enhances the anti-tumor efficacy of both GPC3 and HPV-associated mRNA vaccines. **(A)** Schematic of treatment grouping and administration in a therapeutic subcutaneous B16-hGPC3 tumor-bearing mouse model (n=9). **(B)** Subcutaneous tumor volume curves of mice in various treatment groups. **(C)** Kaplan-Meier overall survival curves of mice in each treatment group. **(D)** Schematic of treatment grouping and administration in a therapeutic subcutaneous H658-related tumor-bearing mouse model (n=9). **(E, F)** Subcutaneous tumor volume curves of B16F10-H658 **(E)** and TC-1 **(F)** mice in various treatment groups. Data are presented as mean ± SEM for tumor volume and body weight curves. *P < 0.05, **P < 0.01, ***P < 0.001, ****P < 0.0001.

For safety evaluation, no significant differences in body weight were observed among all treatment groups throughout the experiment, indicating that the GPC3 mRNA vaccine combined with mXCL1-mCD40L adjuvant was well tolerated with no overt systemic toxicity (**Supplementary Figure 10B**).

The tumor growth curves demonstrated that the GPC3+mXCL1-mCD40L group exhibited the most potent tumor growth inhibition among the treatment groups, with a significantly slower tumor growth rate (13.72 ± 3.94 mm³/day) than the GPC3+Luc group (34.91 ± 4.09 mm³/day, *P* = 3.8 × 10^-4^). The anti-tumor effect of GPC3+mXCL1-mCD40L was also significantly stronger than that of the GPC3+mCD40L mono-adjuvant group (29.78 ± 4.07 mm³/day, *P* = 0.0046, **Figure 4B**). Individual tumor growth curves and AUC analysis are presented in **Supplementary Figures 10C, D**. Kaplan-Meier survival analysis showed that compared with the Luc control group, all GPC3-treated groups significantly prolonged the survival of tumor-bearing mice (*P* < 0.0001, **Figure 4C**).

Notably, the GPC3+mXCL1 mono-adjuvant group unexpectedly showed a trend toward increased tumor growth compared with the GPC3+Luc group. This unexpected observation suggests that cDC1-oriented chemotactic signaling alone may be insufficient to generate efficient anti-tumor immunity in the absence of simultaneous maturation signals. However, the underlying mechanism requires further investigation.

The generalizability of a cancer vaccine adjuvant across distinct tumor types and antigen targets is critical for evaluating its broader translational potential. To evaluate whether mXCL1-mCD40L mRNA-LNP could enhance the efficacy of mRNA vaccines beyond the GPC3 antigen setting, we designed an mRNA vaccine encoding the E6/E7 proteins of HPV16, HPV18, and HPV52 (H658). We established early therapeutic tumor-bearing mouse models using TC-1 cells and B16F10-H658 cells, as described in the Methods section. Treatment was initiated on day 3 after tumor inoculation (**Figure 4D**). Quantitative RT-PCR confirmed that B16F10-H658 related cell lines stably expressed E6/E7 mRNA (**Supplementary Figures 10A** Right).

The results showed that, compared with the H658 mRNA-LNP monotherapy group, the combination treatment group (H658 mRNA-LNP plus mXCL1-mCD40L mRNA-LNP) displayed significantly better tumor growth inhibition in both TC-1 and B16F10-H658 tumor-bearing mouse models (**Figures 4E, F**). Individual tumor growth curves and per-mouse AUC analyses are provided in **Supplementary Figure 11**. These results suggest that mXCL1-mCD40L mRNA-LNP can enhance mRNA vaccine efficacy in an additional HPV-associated antigen setting, supporting its potential as an antigen-flexible mRNA vaccine adjuvant platform.

### 3.5 The mXCL1-mCD40L fusion mRNA-LNP enhances anti-tumor efficacy over co-delivery of individual adjuvant mRNAs in tumor-bearing mouse models

To compare the therapeutic efficacy of the fusion design with co-delivered individual adjuvant, we established a B16F10-hGPC3 subcutaneous tumor model in C57BL/6 mice. (**Figure 5A**).

**Figure 5:**
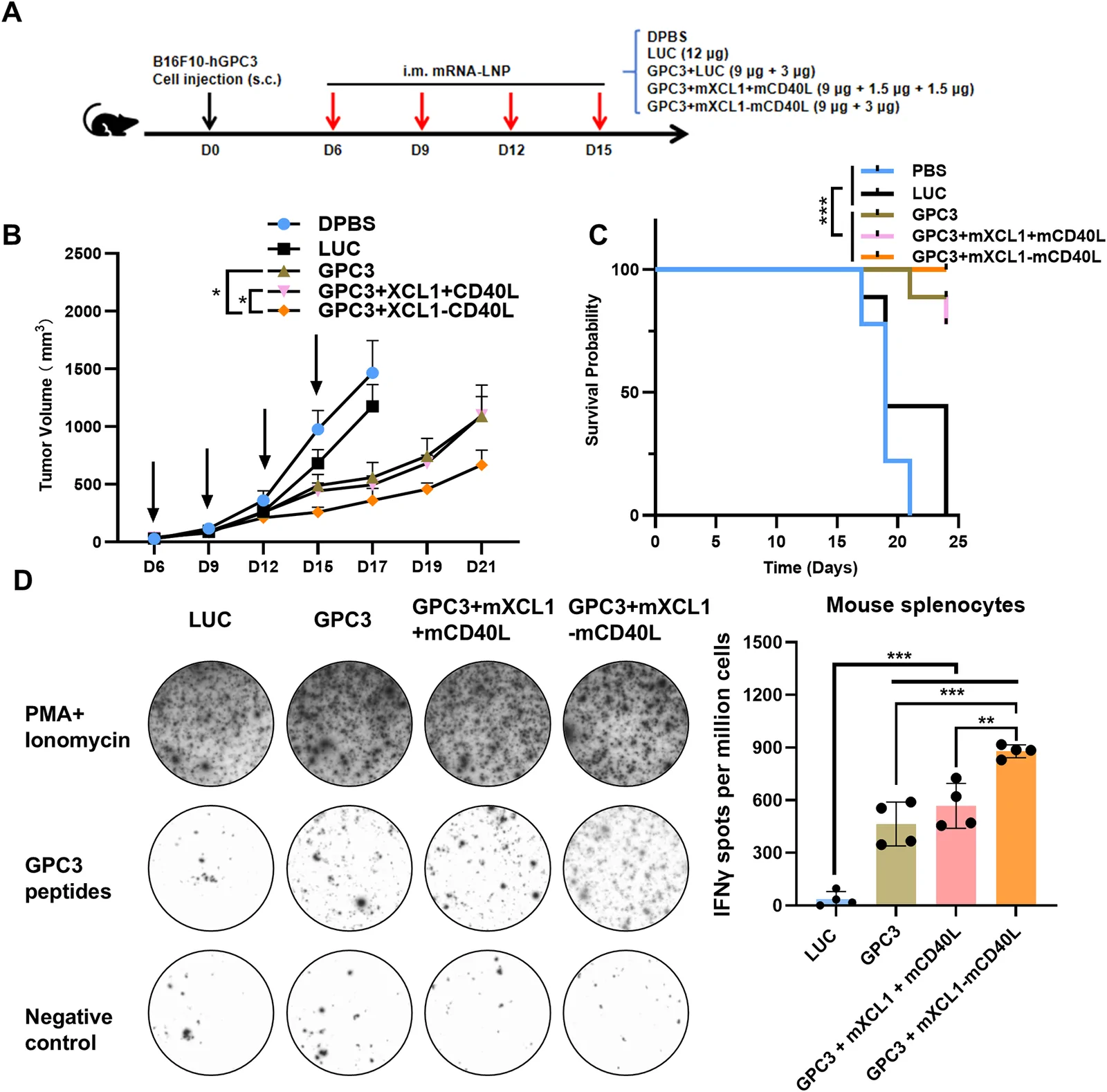
mXCL1-mCD40L fusion mRNA-LNP confers superior anti-tumor efficacy compared with co-delivery of separate mXCL1 and mCD40L adjuvant mRNAs in B16F10-hGPC3 tumor-bearing mice. **(A)** Schematic of treatment grouping and administration in a therapeutic subcutaneous tumor-bearing mouse model (n=9). **(B)** Subcutaneous tumor volume curves of mice in various treatment groups. **(C)** Kaplan-Meier overall survival curves of mice in each treatment group. **(D)** Representative images and quantitative summary of IFN-γ ELISPOT assays. Isolated mouse splenocytes were restimulated with GPC3 peptides, PMA+Ionomycin (positive control), or medium negative control. Data are presented as mean ± SEM for tumor volume curves. *P < 0.05, **P < 0.01, ***P < 0.001.

Whereas tumors in the DPBS and Luc groups grew rapidly, GPC3 vaccination alone only modestly delayed tumor growth. Linear mixed-effects growth-rate modeling revealed significant differences among the GPC3-containing groups (*P* = 0.0007): the fusion group grew significantly more slowly than the GPC3 group (*P* = 0.0018), whereas the co-delivery group did not differ from GPC3 alone (*P* = 0.92); the direct fusion showed significantly better efficacy than the co-delivery group (*P* = 0.0018) (**Figure 5B**). Individual tumor growth curves and per-mouse AUC analyses are provided in **Supplementary Figure 12**. Kaplan-Meier survival analysis showed that all GPC3-treated groups exhibited significantly improved survival compared with the control group (*P* < 0.001) (**Figure 5C**).

To determine whether the superior tumor control was accompanied by enhanced antigen-specific T-cell priming, we performed IFN-γ ELISPOT assays using splenocytes restimulated with GPC3 peptides. GPC3 vaccination markedly increased GPC3-specific IFN-γ responses relative to the Luc control (464 ± 121 vs. 37 ± 42 spot-forming cells per 10^6^ splenocytes, *P* < 0.001). Notably, the fusion regimen further amplified this response (879 ± 37 SFC/10^6^; vs. GPC3 alone, 465 ± 125, *P* < 0.001; vs. co-delivery, 568 ± 128 SFC/10^6^, *P* = 0.003), whereas co-delivery did not significantly enhance responses over GPC3 alone (*P* = 0.44) (**Figure 5D**).

Together, these results demonstrate that, at matched adjuvant and total mRNA doses, the single-molecule mXCL1-mCD40L fusion design confers significantly greater tumor growth control and antigen-specific T-cell priming than co-delivery of separate mXCL1 and mCD40L mRNAs, establishing the necessity of physically coupling the recruitment and maturation functions within one molecule.

### 3.6 mXCL1-mCD40L mRNA-LNP promotes antigen acquisition and presentation

To directly track antigen uptake *in vivo*, we established a B16F10-GFP subcutaneous tumor-bearing model, employing GFP as a tracer antigen to detect tumor-antigen acquisition by cDC1s in regional dLNs (**Figure 6A**). Flow cytometry analysis revealed that the proportion of GFP^+^ cells within the CD11c^+^CD103^+^ cDC1 population in the dLNs was significantly higher in the mXCL1-mCD40L fusion mRNA group than in the Luc control group (*P* < 0.001). Compared with the mXCL1+mCD40L co-delivery group, the mXCL1-mCD40L fusion mRNA group exhibited a significantly greater increase in GFP^+^ cells within the CD11c^+^CD103^+^ cDC1 population in the dLNs (*P* < 0.01) (**Figure 6B**). In mice bearing GFP-negative B16F10-WT tumors treated with mXCL1-mCD40L, GFP^+^ cDC1 remained at background levels, confirming that the GFP signal reflects genuine tumor-antigen acquisition rather than autofluorescence. These data indicate that the fusion mRNA promotes tumor-antigen acquisition by cDC1 more efficiently than co-delivery of the two separate mRNAs.

**Figure 6:**
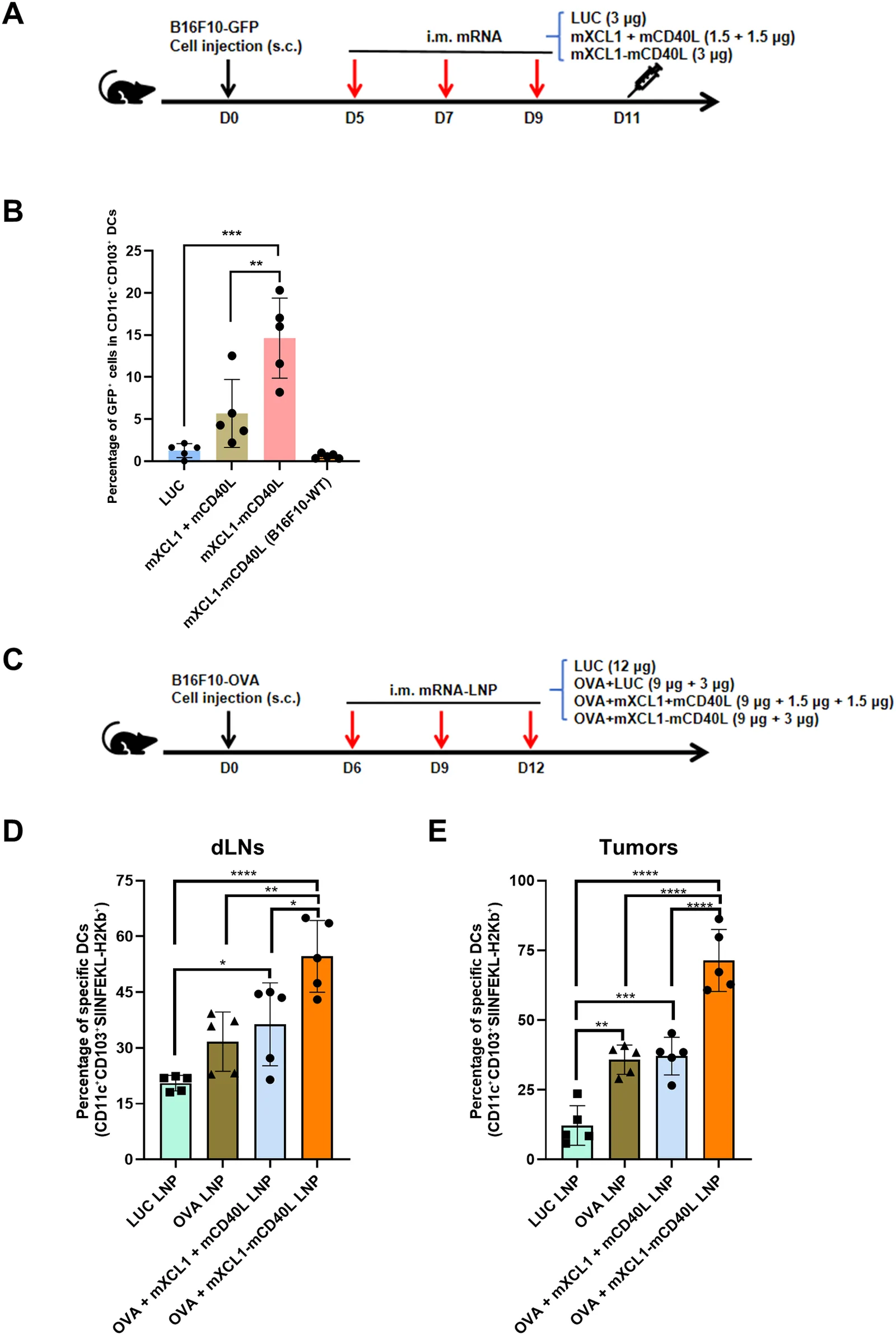
The mXCL1-mCD40L fusion mRNA adjuvant promotes tumor antigen uptake and antigen presentation by CD103^+^ DCs. **(A)** Schematic of the experimental design for assessing tumor antigen uptake by cDC1s in tumor dLNs. **(B)** Quantification of the proportion of GFP^+^ cells in CD11c^+^CD103^+^ cDC1 in the dLNs of mice in each group. **(C)** Schematic of the experimental design for evaluating antigen-specific DC presentation in the B16-OVA tumor model. **(D)** Flow cytometric quantification of antigen-specific DCs (CD11c^+^CD103^+^SIINFEKL–H-2Kb^+^) in dLNs. **(E)** Flow cytometric quantification of antigen-specific DCs (CD11c^+^CD103^+^SIINFEKL–H-2Kb^+^) in tumor tissues. Data are presented as mean ± SEM. *P < 0.05, **P < 0.01, ***P < 0.001, ****P < 0.0001.

To evaluate whether the mXCL1-mCD40L adjuvant enhances tumor antigen presentation by CD103^+^ DCs, we established a subcutaneous B16-OVA tumor model, in which the MHC I–restricted OVA epitope SIINFEKL permits direct tracking of antigen-specific presentation. As illustrated in **Figure 6C**, mice were inoculated with B16-OVA tumor cells on Day 0 and received intramuscular injections of the mRNA-LNP formulations on Days 6, 9, and 12. On Day 14, dLNs and tumors were harvested for flow cytometric quantification of CD11c^+^CD103^+^SIINFEKL–H-2K^b+^ antigen-specific DCs.

In the dLNs (**Figure 6D**), the co-delivered mXCL1+mCD40L adjuvant increased this population relative to Luc alone (*P* < 0.05). The OVA+mXCL1-mCD40L fusion group showed the highest proportion of antigen-specific DCs of all groups, higher than the OVA-only group (*P* < 0.01) and the co-delivery group (*P* < 0.05).

Antigen-specific DCs infiltration showed a consistent pattern in the tumor. (**Figure 6E**). Tumor-infiltrating antigen-specific DCs were significantly increased in the OVA group than in the Luc control (*P* < 0.01), and the co-delivery adjuvant failed to increase the percentage of antigen-specific DCs. In contrast, the mXCL1-mCD40L fusion adjuvant markedly enhanced tumor infiltration of antigen-specific DCs, with levels significantly higher than both the OVA-only group (*P* < 0.001) and the co-delivery group (*P* < 0.0001).

Together, these data show that the mXCL1-mCD40L fusion mRNA adjuvant enhances tumor antigen presentation by CD103^+^ DCs both in the regional dLNs and in the tumor, which may contribute to improved initiation of anti-tumor immune responses.

### 3.7 mXCL1-mCD40L mRNA-LNP by intramuscular administration shows local biodistribution and enhanced anti-tumor efficacy relative to intratumoral delivery

To characterize the biodistribution of intramuscularly injected mRNA-LNP, we first performed *in vivo* bioluminescence imaging with a luciferase-encoding construct. The signal was confined to the injection site across lateral, dorsal, and ventral views, with only minimal signal in distant organs (**Figure 7A**). Expression of the encoded protein delivered by SM102 LNP system was largely local distribution.

**Figure 7:**
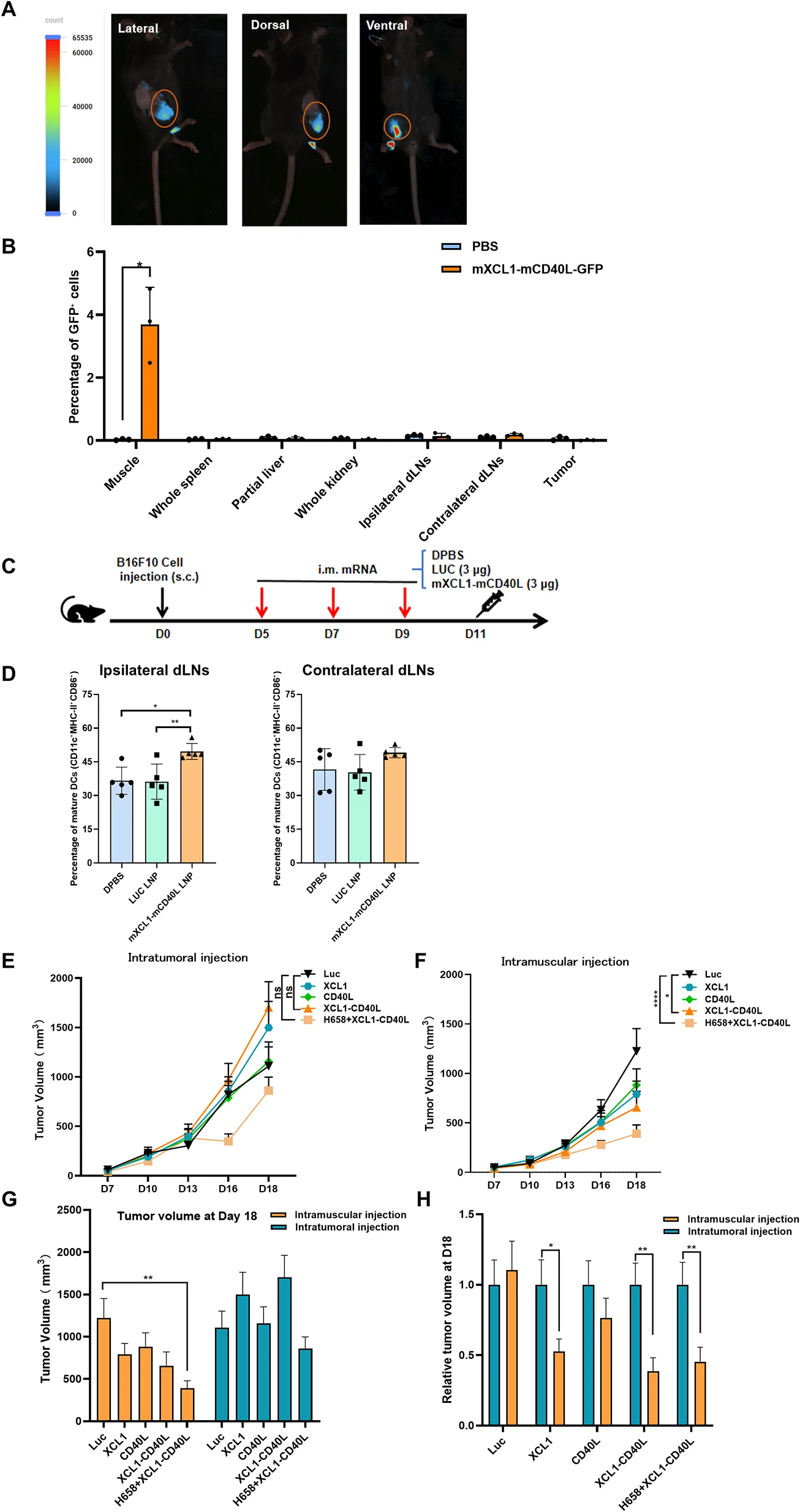
Biodistribution, regional DC maturation and route-dependent anti-tumor efficacy of intramuscular mXCL1-mCD40L mRNA-LNP. **(A)** Bioluminescence imaging of mice at 4 h after intramuscular injection of Luc mRNA-LNP, presented in lateral, dorsal and ventral views. The bioluminescent signal is largely confined to the injection site. **(B)** Flow cytometric quantification of the percentage of GFP^+^ cells across different tissues at 16 h after intramuscular administration of mXCL1-mCD40L-GFP mRNA-LNP. PBS served as the control. **(C)** Schematic of the experimental design for assessing DC maturation in ipsilateral versus contralateral dLNs. **(D)** Flow cytometric quantification of mature DCs (CD11c^+^MHC-II^+^CD86^+^) in ipsilateral (left) and contralateral (right) dLNs. **(E, F)** Tumor growth curves of B16F10-H658 tumor-bearing mice treated with the indicated formulations via intratumoral **(E)** or intramuscular **(F)** injection on days 7, 10, 13, and 16 (n = 9). **(G, H)** Quantification of absolute tumor volume **(G)** and relative tumor volume **(H)** on day 18, comparing the therapeutic effects between intratumoral and intramuscular administration routes. Data are presented as mean ± SEM. The results were assessed by one-way ANOVA followed by Tukey’s multiple comparison test. *P < 0.05, **P < 0.01.

We next examined this distribution at the cellular level with a GFP reporter system. Mice bearing subcutaneous B16F10 tumors received an intramuscular injection of 20 μg mXCL1-mCD40L-GFP mRNA-LNP. The GFP moiety was fused to the C-terminus of mXCL1-mCD40L via a GS linker, and correct expression of the mXCL1-mCD40L-GFP fusion protein was validated in B16F10 cells *in vitro* (**Supplementary Figure 13**). Sixteen hours later, we harvested the injection-site muscle, bilateral dLNs, spleen, kidney, tumor, and part of the liver, and quantified GFP^+^ cells by flow cytometry. Consistent with the imaging data, GFP^+^ cells were almost entirely confined to the injection-site muscle (**Figure 7B**). Intramuscularly delivered mRNA-LNP thus drives protein expression locally, with limited systemic dissemination.

To evaluate the regional effect on DC-maturation of mXCL1-mCD40L mRNA-LNP, we established a subcutaneous wild-type B16F10 tumor model by administrated intramuscularly mRNA-LNP and assessed DC maturation in ipsilateral versus contralateral dLNs (**Figure 7C**). In the ipsilateral dLNs, the mXCL1-mCD40L group showed a significantly higher percentage of mature DCs than the DPBS group (*P* < 0.05) and the Luc group (*P* < 0.01). Mature DCs rose from 30–40% in the two control groups to approximately 50% with the fusion adjuvant. However, none of the groups differed significantly in the contralateral dLNs (**Figure 7D**). Intramuscular mXCL1-mCD40L mRNA-LNP thus promotes DC maturation locally in the ipsilateral dLNs, without significant systemic effect in the contralateral dLNs.

We next compared the anti-tumor efficacy of the mXCL1-mCD40L adjuvant between intratumoral and intramuscular administration (**Figures 7E, F**). After intratumoral injection, neither the single adjuvant nor the antigen–adjuvant combinations appreciably inhibited tumor growth. After intramuscular injection, both mXCL1-mCD40L monotherapy (*P* < 0.05) and the H658+mXCL1-mCD40L (*P* < 0.0001) combination significantly suppressed tumor growth relative to the Luc control. To further quantify the administration route-dependent therapeutic difference, we compared absolute and relative tumor volumes on day 18 across all treatment groups (**Figures 7G, H**). For the intratumoral administration route, none of the groups showed significant tumor volume reduction compared with the Luc control on day 18. For the intramuscular administration, the H658+mXCL1-mCD40L combination achieved measurable tumor growth inhibition relative to the Luc control. A head-to-head comparison of the two administration routes demonstrated that therapeutic mRNA-LNPs yielded smaller relative tumor volumes via intramuscular delivery versus intratumoral injection. Thus, the anti-tumor efficacy of the mXCL1-mCD40L mRNA-LNP is route-dependent in this model, with intramuscular administration outperforming direct intratumoral delivery.

To assess the safety and translational feasibility of the GPC3+mXCL1-mCD40L combination, we measured serum ALT, AST, and IL-12 after the first intramuscular dose and circulating CD8^+^ T cells on day 11 (**Supplementary Figures 14A–D**). Liver enzymes and IL-12 did not differ among the four groups at 4 or 24 h, whereas the combination increased the peripheral blood CD8^+^ T cell proportion above all three controls (*P* < 0.0001 vs DPBS; *P* < 0.001 vs Luc and GPC3+Luc). Lyophilized mXCL1-mCD40L mRNA-LNP retained this CD8^+^ T cell-inducing activity after reconstitution (**Supplementary Figures 14E, F**).

## 4 Discussion

In this study, we addressed an important challenge in cancer immunotherapy: impaired cDC1 functional activation and insufficient cDC1-oriented immune-activating signals within the TME (10, 33). This defect represents a key bottleneck limiting the anti-tumor efficacy of mRNA vaccines. Based on clinical transcriptomic evidence from HCC and melanoma, we developed a novel dual-function mRNA adjuvant encoding mXCL1-mCD40L, which significantly enhanced the anti-tumor efficacy of the GPC3 mRNA vaccine and HPV-related therapeutic mRNA vaccine. This study provides a rational technical strategy to alleviate the efficacy bottleneck of mRNA vaccines against solid tumors.

The functional state of cDC1 is a critical determinant of the magnitude of endogenous anti-tumor immune responses (34, 35). Previous studies have mostly focused on the correlation between cDC1 infiltration abundance and clinical prognosis in individual cancer types (11, 35). Targeting cDC1s represents an important direction for next-generation cancer vaccine adjuvant development (36, 37). However, strategies that enhance either chemotaxis or immune activation alone may be insufficient in immunosuppressive solid tumors (11, 38). Our TCGA analysis extended these observations, showing that a cDC1 signature score stratified patients by overall survival in both the LIHC and SKCM cohorts. Transcriptomic analysis revealed that XCL1-XCR1 signaling in HCC and CD40-CD40L signaling in melanoma were respectively downregulated. Given that distinct tumors harbor differential defects in either XCL1-mediated cell recruitment or CD40-mediated maturation signaling, a fusion strategy integrating the functions of both XCL1 and CD40 can maximize anti-tumor activity by restoring both signaling inputs, irrespective of which axis constitutes the limiting step in a given tumor.

Our *in vivo* data support this concept: mXCL1 alone did not improve the therapeutic efficacy of the GPC3 mRNA vaccine, whereas the mXCL1-mCD40L fusion adjuvant produced stronger tumor control than either single-function adjuvant. These findings suggest that cDC1-directed chemotactic signaling needs to be coupled with maturation signaling to generate stronger anti-tumor immunity. Consistent with this coupling requirement, the fusion adjuvant restored antigen cross-presentation by CD103^+^ DCs in both dLNs and tumors in the B16-OVA model.

From an engineering perspective, encoding both functional modules within a single fusion mRNA may reduce temporal and spatial mismatch compared to co-delivery of both functional modules by two independent mRNAs (39). Functional validation of the mXCL1-mCD40L fusion protein, encompassing expression, secretion, chemotactic activity, and DC maturation capacity, confirms the efficacy and rationality of this single fusion design. In addition, the fusion-mRNA strategy may simplify formulation development and quality control compared with multi-mRNA adjuvant combinations. Relative to co-delivery of individual mXCL1 and mCD40L mRNA-LNP at equal total adjuvant doses, the single-molecule fusion design enhanced tumor growth controlling and antigen-specific T-cell priming. The benefit likely arises from synchronized biodistribution and matched expression kinetics for both moieties within one mRNA. Clinical translatability is a core consideration in the development of mRNA-based cancer vaccines. Our safety assessment demonstrated that the combination regimen of GPC3 mRNA vaccine plus mXCL1-mCD40L adjuvant had no detectable risk of acute hepatotoxicity or systemic IL-12 response in mouse peripheral blood. In parallel, we found that reconstituted solutions of lyophilized mRNA-LNP maintained equivalent CD8^+^ T cell-inducing activity compared to the liquid formulations. This finding provides preliminary support for further development of storage-compatible mRNA-LNP formulations and may help reduce cold-chain dependence after additional stability optimization.

Several limitations of this study should be pointed out. Our findings were generated primarily in immunocompetent mouse tumor models, and their relevance to human immune biology requires further validation. Second, B16F10-hGPC3 is an engineered model, and the xenogeneic human GPC3 may exhibit extra immunogenicity. Validation in endogenous GPC3^+^ models is warranted. Third, we used dose-matched Luc mRNA-LNP as an irrelevant mRNA control with identical mRNA chemistry and transcript length. Nevertheless, luciferase is not completely immunologically inert, and our study did not include baseline PBS and empty LNP controls, which would have strengthened the experiment results. Fourth, bioluminescence imaging based on Luc mRNA predominantly reflects the delivery location of SM102 LNP system and may not perfectly recapitulate the distribution of the fusion construct, although cellular-level mapping with an mXCL1-mCD40L-GFP reporter confirmed local restriction. In addition, the HPV-bearing tumor models lacked single-agent and pooled co-delivery adjuvant control groups. We will carry out these validations in future work.

Although previous studies have demonstrated that human and murine cDC1s are highly conserved in their development and antigen-presenting functions (40, 41), the expression profile of XCR1 in human immune cell subsets, and the chemotactic activity of human XCL1 toward primary human cDC1, remain to be validated in clinical tumor specimens. Thus, future optimization may combine our adjuvanted vaccine with immune checkpoint inhibitors, serving as a promising approach to reverse TME immunosuppression and improve anti-tumor efficacy. Finally, to address the intrinsic heterogeneity of solid tumors, multi-antigen in personalized neoantigen vaccine is promising strategy that has been clinically validated (42), and our dual-functional adjuvant platform can be readily adapted to these pipelines.

In summary, we have developed an efficient and well-tolerated dual-functional mRNA adjuvant platform. This work not only provides an innovative strategy to overcome the efficacy bottleneck of mRNA cancer vaccines against solid tumors, but also establishes a feasible design framework for cancer immunotherapies targeting the functional restoration of cDC1.

## Data Availability

The original contributions presented in the study are included in the article/**Supplementary Material**. Further inquiries can be directed to the corresponding authors.
