# Supplementary material for: A dual-function XCL1-CD40L mRNA adjuvant potentiates cancer vaccines by coordinating DC recruitment and maturation
Source: Front Immunol. 2026 Oct 14;17:1875603. doi: 10.3389/fimmu.2026.1875603 (PMC13713712; doi:10.3389/fimmu.2026.1875603)
Supplement: Supplementary file 1 [file DataSheet1.pdf]

Supplementary Table 1 Major cell cluster annotation markers

Gene	marker
Pan-Leukocytes	*PTPRC*
T Lymphocytes	*CD3D, CD3E, CD8A, CD4*
NK Cells	*KLRD1, GNLY, NKG7, NCAM1*
Myeloid Cells	*CD68, CD163, CD14, LYZ, ITGAX, FCGR3A*
B Lymphocytes	*MS4A1, CD79A*
Plasma Cells	*MZB1, JCHAIN*
Endothelial Cells	*PECAM1, VWF, CLDN5*
Fibroblasts	*ACTA2, COL1A1, COL1A2, DCN, LUM*
Mast Cells	*TPSAB1, CPA3*
Hepatocytes/Tumor	*ALB, APOA1，AFP, GPC3, EPCAM, KRT19, KRT7*
Lung Epithelial Cells/Tumor Cells	*SFTPC，AGER，EPCAM, KRT7, KRT18, KRT19，NKX2-1, NAPSA*
Malignant Melanoma Cells	*PMEL, MLANA, TYR, MITF*

Supplementary Table 2 Cell subpopulation annotation markers

Gene	marker
T Lymphocytes	*CD3D, CD3E, CD4, CD8*
NK Cells	*KLRD1, GNLY, NKG7, NCAM1*
Effector/Cytotoxic CD8+ T Cells	*CD8A, CD8B, GZMB, PRF1, IFNG, NKG7, NCAM1, FCGR3A, KLRD1, KLRF1, GNLY*
Exhausted CD8+ T Cells	*PDCD1, LAG3, HAVCR2, TOX*
Memory/Naive CD8+ T Cells	*TCF7, LEF1, SELL*
Tregs	*FOXP3, IL2RA, CTLA4*
Naive/Central Memory CD4+ T Cells	*IL7R, CCR7*
Tfh	*CXCL13*
Proliferating T Cells	*MKI67*
pDC	*CLEC4C, IRF7, TCF4, GZMB*
cDC1	*XCR1, CLNK, CLEC9A*
cDC2	*FCER1A, CD1E, CD1C, CLEC10A*
LAMP3+ DC	*CCL19, LAMP3, CCR7, LY75, CCL22,*

	NFKB2
C1Q+ TAM	*APOC1, HLA-DRB5, C1QA, C1QB, CD68, CD163*
LYVE1+ Tissue-Resident Macrophages	*THBS1, FOLR2, LYVE1, MRC1*
SPP1+ TAM	*APOE, TREM2, SPP1, CD9*
Monocytes	*TIMP1, S100A11, CXCL8, IL1B, PTGS2, S100A9, S100A8, MMP19*

Supplementary Table 3 Characterization of mRNA-LNP

mRNA-LNP	source	EE%	particle size (nm)	PDI
Luc	Fig 2	95.51%	122.47	0.124
mXCL1-mCD40L	Fig 2	95.55%	117.82	0.106
Luc	Fig 4, 7D, Sup Fig15	96.50%	115.68	0.127
mXCL1	Fig 4, 7D, Sup Fig15	96.00%	117.46	0.125
mCD40L	Fig 4, 7D, Sup Fig15	96.38%	119.35	0.117
mXCL1-mCD40L	Fig 4, 7D, Sup Fig15	96.71%	121.06	0.103
GPC3	Fig 4, 7D, Sup Fig15	94.31%	115.24	0.112
Luc	Fig 3, 5, 6B, D-E 7A	96.51%	125.08	0.081
mXCL1	Fig 3, 5, 6B, D-E 7E-F	96.33%	114.13	0.124
mCD40L	Fig 3, 5, 6B, D-E 7E-F	92.48%	119.65	0.075
mXCL1-mCD40L	Fig 3, 5, 6B, 7E-F	95.17%	111.24	0.132
GPC3	Fig 5	94.47%	124.29	0.115
Luc	pre-lyophilization	95.24%	117.25	0.139
GPC3	pre-lyophilization	94.54%	109.34	0.129
mXCL1-mCD40L	pre-lyophilization	94.72%	110.51	0.122
Luc	post-lyophilization	90.23%	124.62	0.195
GPC3	post-lyophilization	89.74%	120.26	0.166
mXCL1-mCD40L	post-lyophilization	89.89%	116.21	0.142

H658	Fig 4, 7	96.17%	127.27	0.117
mXCL1-mCD40L	Fig 6D-E, 7	97.11%	122.36	0.093
GFP-OVA	Fig 6D-E	94.57%	118.94	0.147
mXCL1-mXCD40L-GFP	Fig 7B	91.25%	122.51	0.112

Supplementary Table 4 Primer sequences

Gene	Sequence (5'-3')	
Xcl1	Forward	AGCGGCTGCCAGTTCAAAAA
	Reverse	TTCACCCATTTGGCTTCTGGA
Cd40lg	Forward	GCGGCAAATACCCACAGTTC
	Reverse	CTTGGCTTGCTTCAGTCACG
Nfkb1	Forward	AGAGCAACCAAAACAGAGGG
	Reverse	TTGCAAATTTTGACCTGTGGGT
Nfkb2	Forward	AATGGACAAGACAGCGGGTT
	Reverse	TGTGGGAGAGAAGTCCCCAA
Ccl2	Forward	TGCCCTAAGGTCTTCAGCAC
	Reverse	AAGGCATCACAGTCCGAGTC
Cxcl1	Forward	TGGCTGGGATTCACCTCAAG
	Reverse	CCGTTACTTGGGGACACCTT
Il12b	Forward	TGGAGCACTCCCCATTCCTA
	Reverse	GCCTTTGCATTGGACTTCGG
Il1b	Forward	TGCCACCTTTTGACAGTGATG
	Reverse	AAGGTCCACGGGAAAGACAC
Cd86	Forward	AACTTACGGAAGCACCCACG
	Reverse	CTTTGTAAATGGGCACGGCA
Tnf	Forward	GCAGGTTCTGTCCCTTTCACT
	Reverse	CATCTTTTGGGGGAGTGCCT
Il6	Forward	TCCTTCCTACCCCAATTTCCA
	Reverse	GCACTAGGTTTGCCGAGTAGA
Nos2	Forward	CAGGTCTTTGACGCTCGGAA
	Reverse	CTGGGGGAACACAGTGATGG
Ifng	Forward	GAGGTCAACAACCCACAGGT
	Reverse	GGGACAATCTCTTCCCCACC
Cxcl10	Forward	AAGCTATGTGGAGGTGCGAC
	Reverse	AACCCCTTGGGAAGATGGTG
GPC3	Forward	TTGTTGTTCGCCATGCCAAG
	Reverse	AACCCCTTGGGAAGATGGTG
Gapdh	Forward	CTGTCAAACAATTCATTGA
	Reverse	ATGCCAGTGAGCTTCCCGTTCAG
