# Supplementary material for: A dual-function XCL1-CD40L mRNA adjuvant potentiates cancer vaccines by coordinating DC recruitment and maturation
Source: Front Immunol. 2026 Oct 14;17:1875603. doi: 10.3389/fimmu.2026.1875603 (PMC13713712; doi:10.3389/fimmu.2026.1875603)
Supplement: Supplementary file 2 [file DataSheet2.pdf]

Supplementary Figure 1

A

T7 promoter **5' UTR**

TAATACGACTCACTATAAGGGAATTCACATTTGCTTCTGACACAACTGT
GTTCACTAGCAACCTCAAACAGACACCGCCACCATGAGACTTCTCCT
CCTGACTTTCCTGGGAGTCTGCTGCCTCACCCCATGGGTTGTGGAA **full length**
GGTGTGGGGACTGAAGTCCTAGAAGAGAGTAGCTGTGTGAACTTACA **mXCL1**
AACCCAGCGGCTGCCAGTTCAAAAAATCAAGACCTATATCATCTGGGA
GGGGGCCATGAGAGCTGTAATTTTTGTCACCAAACGAGGACTAAAAA
TTTGTGCTGATCCAGAAGCCAAATGGGTGAAAGCAGCGATCAAGACT
GTGGATGGCAGGGCCAGTACCAGAAAGAACATGGCTGAAACTGTTC
CCACAGGAGCCCAGAGGTCCACCAGCACAGCGATAACCCTGACTGG
GGGGGGGGGGGGCTCCGGAGGAGGGGGGTCGGGCGGGGGGGGC **$(G4S)_3$ linker**
TCGATGATAGAAACATACAGCCAACCTTCCCCCAGATCCGTGGCAAC
TGGACTTCCAGCGAGCATGAAGATTTTTATGTATTTACTTACTGTTTTC
CTTATCACCCAAATGATTGGATCTGTGCTTTTTGCTGTGTATCTTCATA
GAAGATTGGATAAGGTCGAAGAGGAAGTAAACCTTCATGAAGATTTTG
TATTCATAAAAAAGCTAAAGAGATGCAACAAAGGAGAAGGATCTTTATC
CTTGCTGAACTGTGAGGAGATGAGAAGGCAATTTGAAGACCTTGTCA
AGGATATAACGTTAAACAAAGAAGAGAAAAAAGAAAACAGCTTTGAAA **full length**
TGCAAAGAGGTGATGAGGATCCTCAAATTGCAGCACACGTTGTAAGC **mCD40L**
GAAGCCAACAGTAATGCAGCATCCGTTCTACAGTGGGCCAAGAAAGG
ATATTATACCATGAAAAGCAACTTGGTAATGCTTGAAAATGGGAAACAG
CTGACGGTTAAAAGAGAAGGACTCTATTATGTCTACACTCAAGTCACC
TTCTGCTCTAATCGGGAGCCTTCGAGTCAACGCCCATTCATCGTCGG
CCTCTGGCTGAAGCCCAGCAGTGGATCTGAGAGAATCTTACTCAAGG
CGGCAAATACCCACAGTTCCTCCCAGCTTTGCGAGCAGCAGTCTGTT
CACTTGGGCGGAGTGTTTGAATTACAAGCTGGTGCTTCTGTGTTTGT
CAACGTGACTGAAGCAAGCCAAGTGATCCACAGAGTTGGCTTCTCAT
CTTTTGGCTTACTCAAACTCTGATAATAGTCTAGAGCTCGCTTTCTTGC **5' UTR**
TGTCCAATTTCTATTAAAGGTTCCTTTGTTCCCTAAGTCCAACTACTAA
ACTGGGGGATATTATGAAGGGCCTTGAGCATCTGGATTCTGCCTAATA
AAAAACATTTATTTTCATTGCAAAAAAAAAAAAAAAAAAAAAAAAAAAAA
AAAAAAAAAAAAAAAAGAAAAAAAAAAAAAAAAAAAAAAAAAAAAAAAA **poly(A) tail**
AAAAAAAAGAAAAAAAAAAAAAAAAAAAAAAAAAAAAAAAAAAAAAAA
AAA

B

MRLLLLTFLGVCCLTPWVVEGVGTEVLEESSCVNLQTQRLPVQKIKTYII **full length**
WEGAMRAVIFVTKRGLKICADPEAKWVKAAIKTVDGRASTRKNMAETV **mXCL1**
PTGAQRSTSTAITLTGGGGGSGGGGSGGGGSMIETYSQPSPRSVATG
LPASMKIFMYLLTVFLITQMIGSVLFAVYLHRRLDKVEEEVNLHEDFVFIK
KLKRCNKGEGSLSLLNCEEMRRQFEDLVKDITLNKEEKKENSFEMQR **full length**
GDEDPQIAAHVVSEANSNAASVLQWAKKGYYTMKSNLVMLENGKQLT **mCD40L**
VKREGLYYVYTQVTFCSNREPSSQRPFIVGLWLKPSSGSERILLKAANT
HSSSQLCEQQSVHLGGVFELQAGASVFVNVTEASQVIHRVGFSSFGLL
KL*

Supplementary Figure 1. The diagram illustrates the nucleotide (A) and amino acid (B) sequence design of the mXCL1-mCD40L fusion construct. The mXCL1 and mCD40L coding sequences are fused in-frame, with a $(G4S)_3$ linker connecting the two functional domains.

Supplementary Figure 2

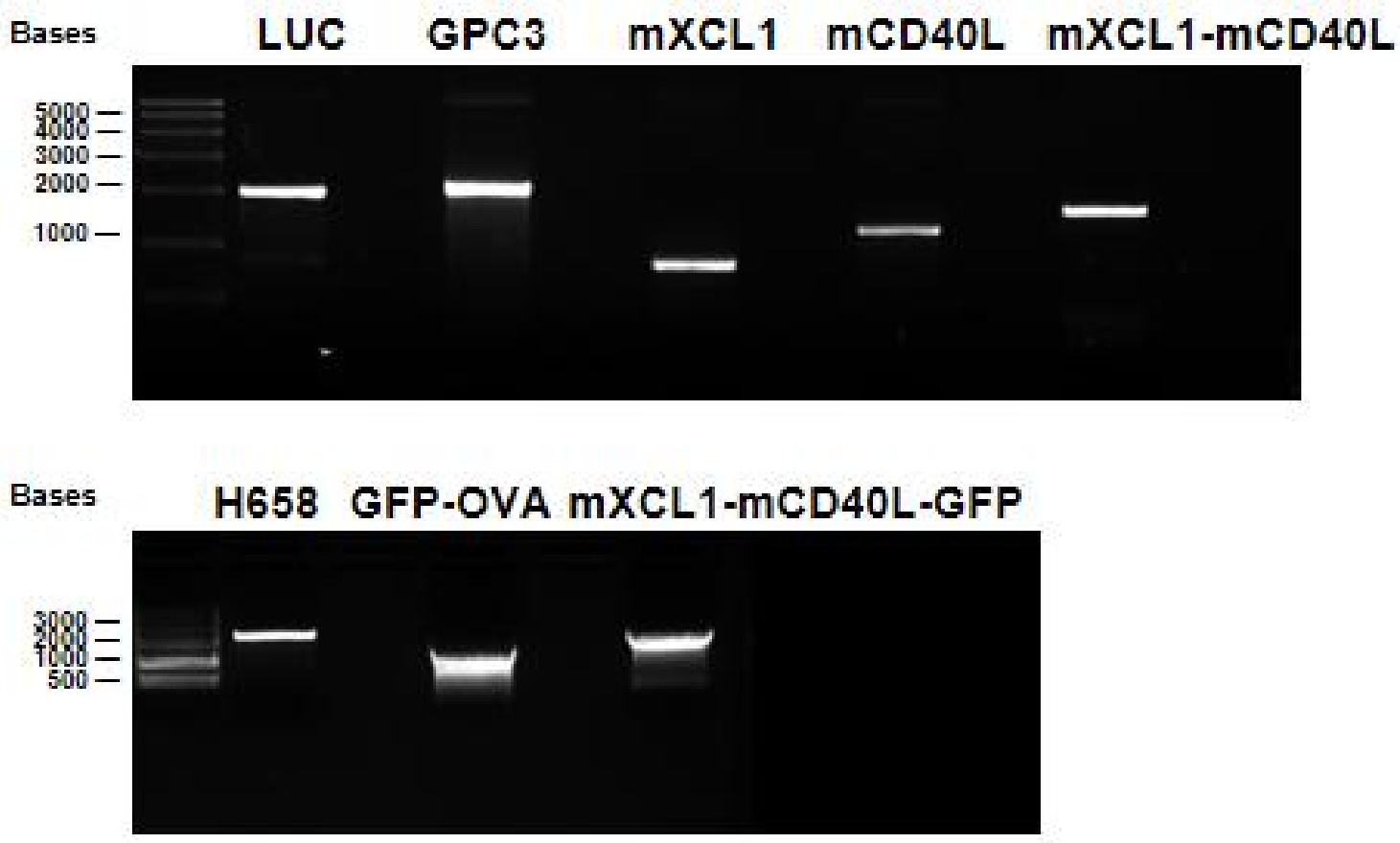

Supplementary Figure 2. Agarose gel electrophoresis analysis of purified in vitro-transcribed mRNAs.

500 ng of each purified mRNA sample was denatured at 65 °C for 10 min and subjected to agarose gel electrophoresis to assess mRNA integrity and molecular size. LUC, GPC3, mXCL1, mCD40L, mXCL1-mCD40L, H658, GFP-OVA, mXCL1-mCD40-GFP mRNA are shown in individual lanes.

Supplementary Figure 3

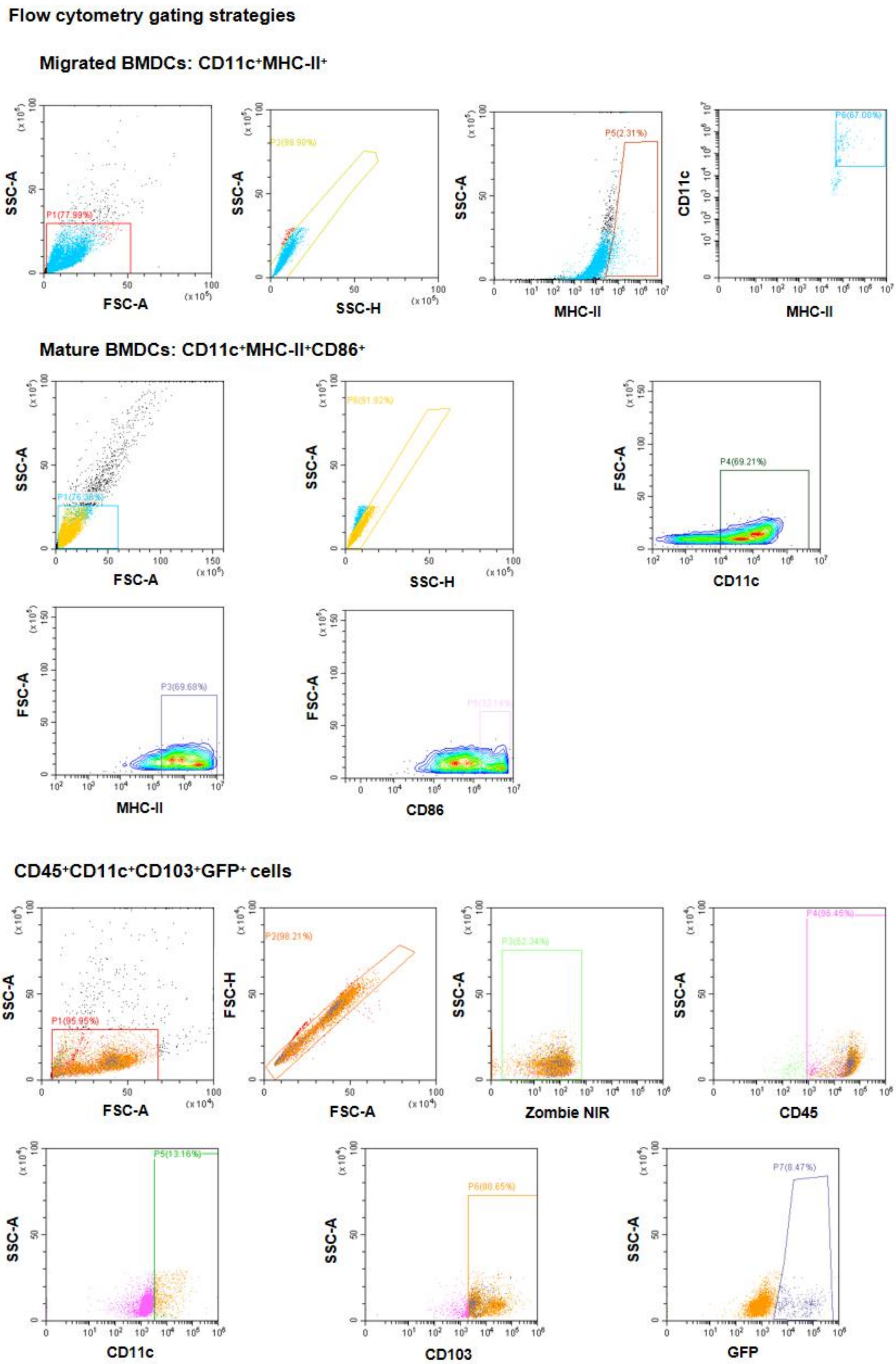

CD45⁺CD11c⁺CD103⁺SIINFEKL–H-2Kᵇ⁺

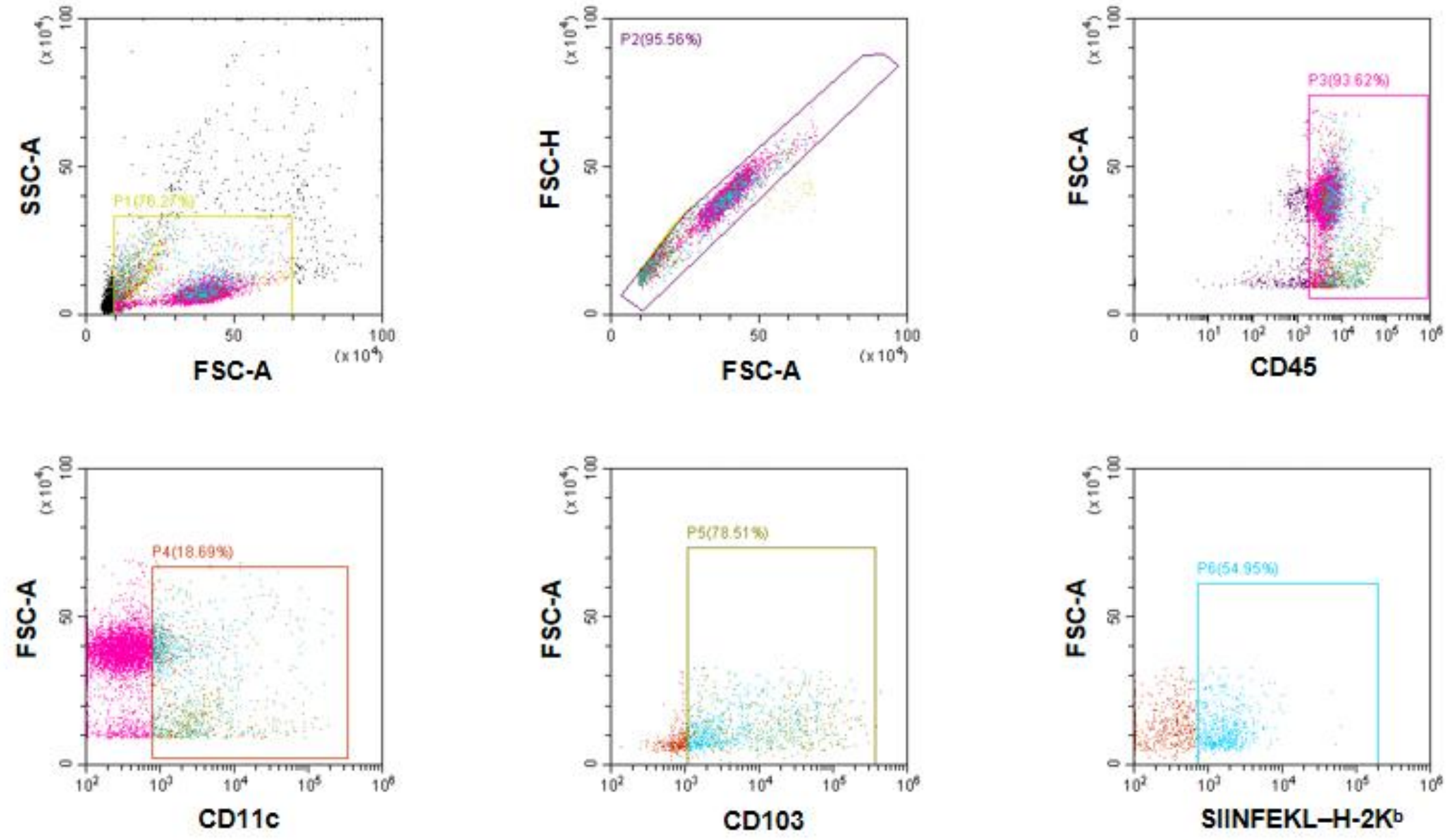

T cells: CD45⁺CD3⁺CD8a⁺

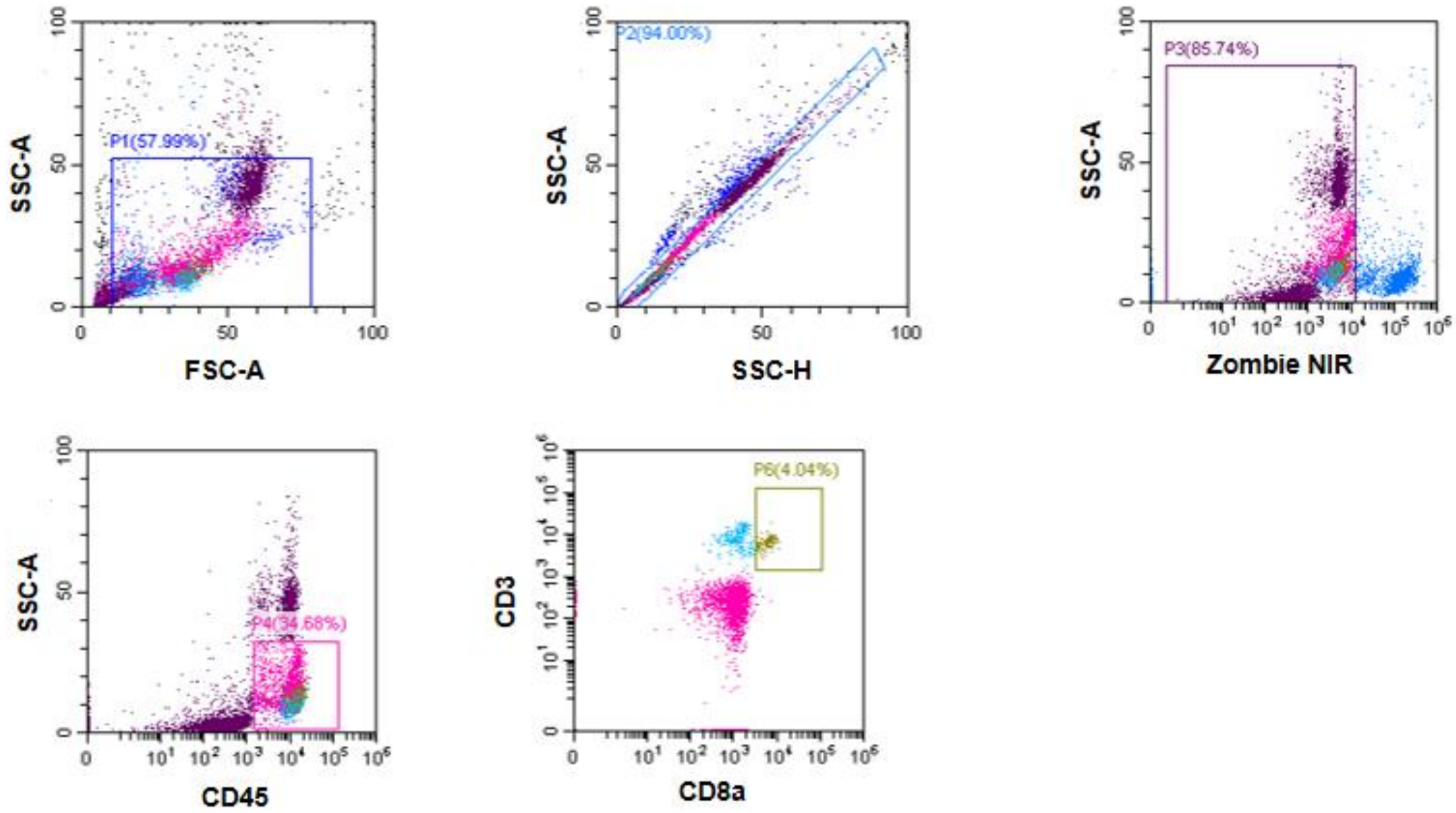

Supplementary Figure 3. Flow cytometry gating strategies applied in this study

Supplementary Figure 4

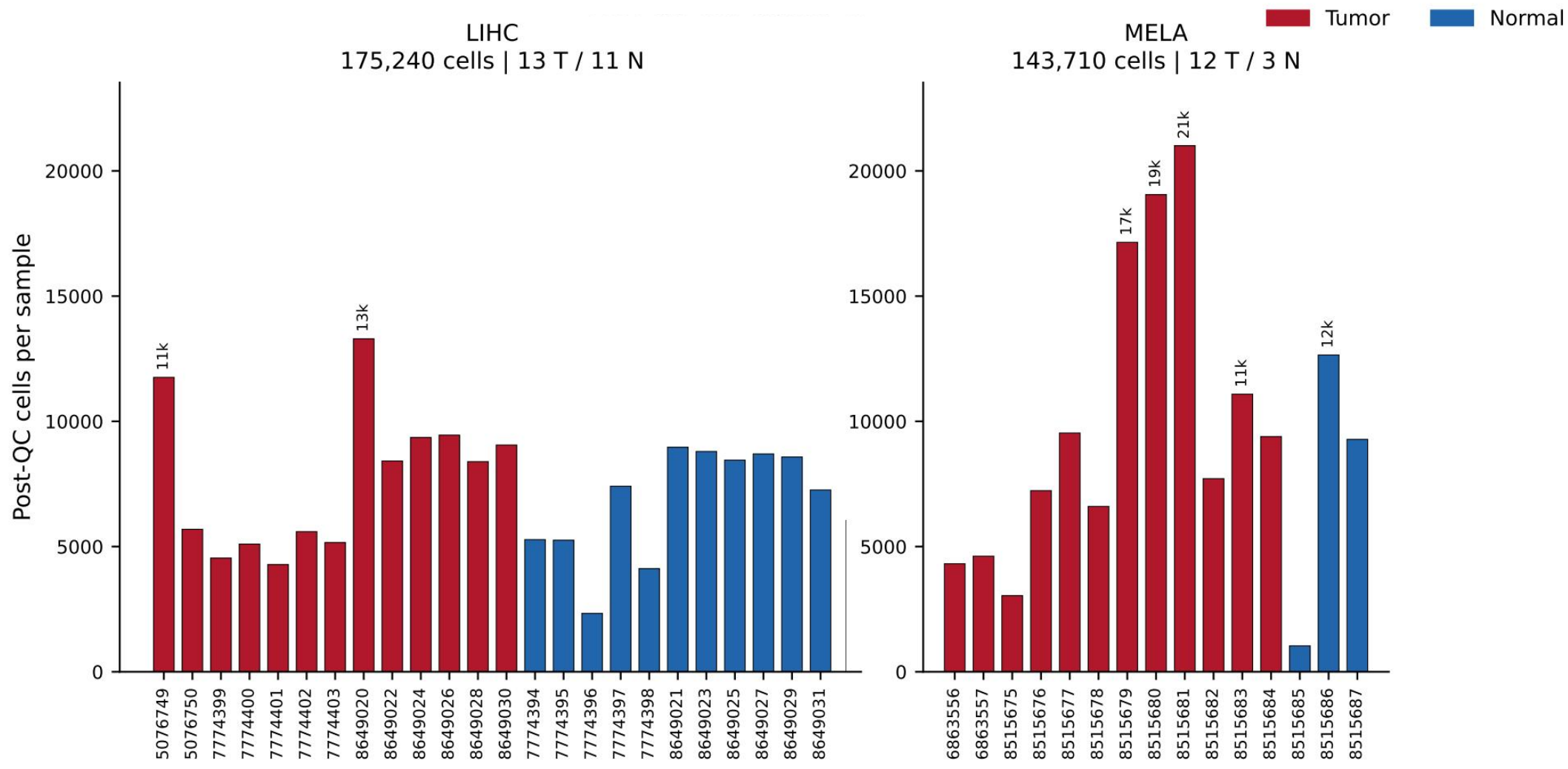

Supplementary Figure 4. Summary of cell numbers retained after quality control across public scRNA-seq datasets. The number of cells retained after quality control filtering is shown for each public single-cell RNA sequencing dataset analyzed in this study. Datasets were categorized into hepatocellular carcinoma (LIHC), melanoma cohorts. Each bar represents one dataset, with the corresponding post-QC cell count labeled above the bar.

Supplementary Figure 5

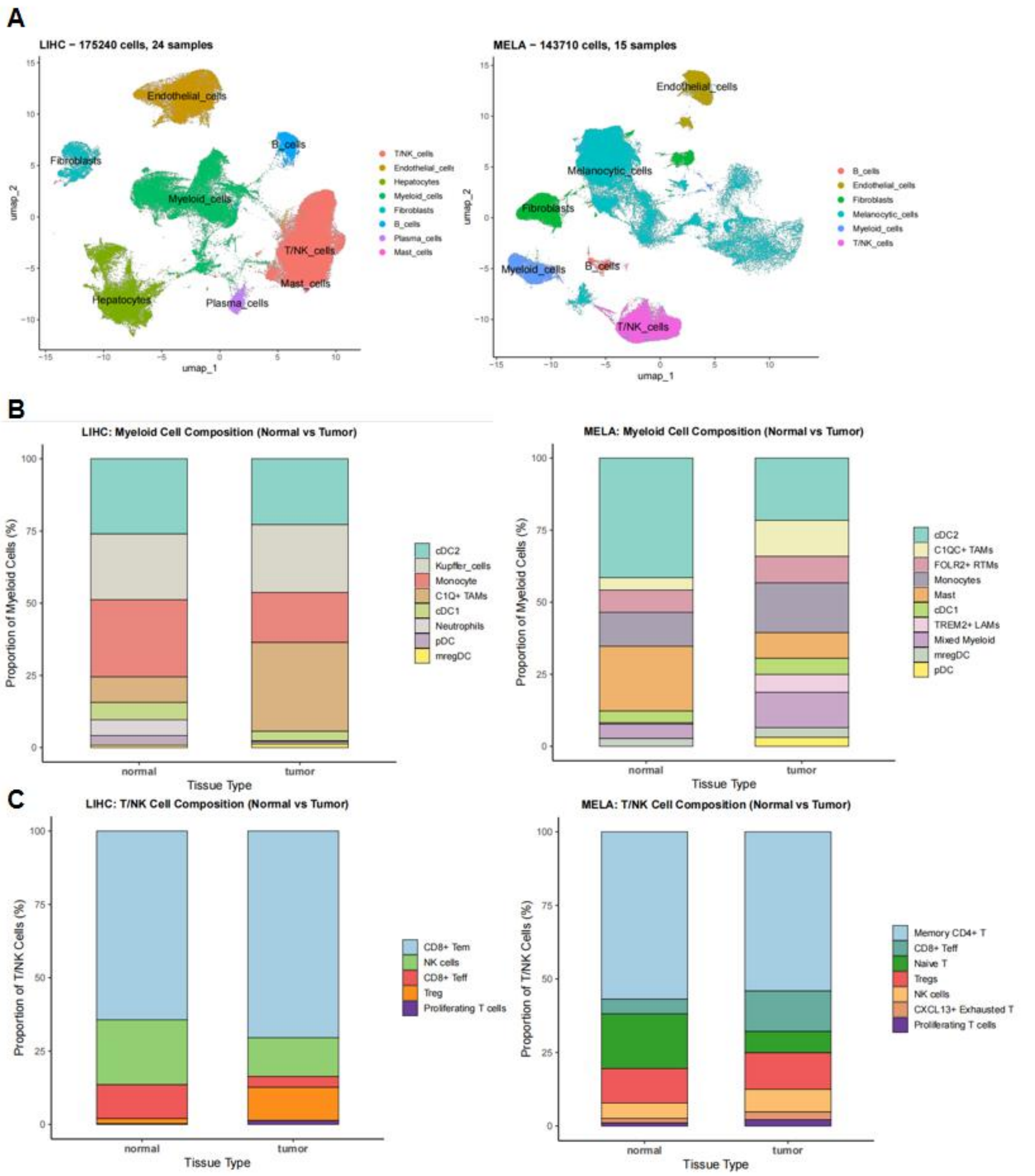

Supplementary Figure 5. Cell type annotation and immune subset composition analysis of single-cell transcriptomes from HCC and melanoma. (A) UMAP visualization of single-cell transcriptomes from the LIHC cohort (left, 175 240 cells from 24 samples) and MEL cohort (right, 143 710 cells from 15 samples), annotated with major cell types including T/NK cells, myeloid cells, epithelial cells/hepatocytes, B cells, plasma cells, fibroblasts, and mast cells. (B) Stacked bar charts comparing the proportional composition of myeloid cell subsets between normal and tumor tissues in the LIHC (left) and MEL (right) cohorts. (C) Stacked bar charts comparing the proportional composition of T/NK cell subsets between normal and tumor tissues in the LIHC (left) and MEL (right) cohorts.

Supplementary Figure 6

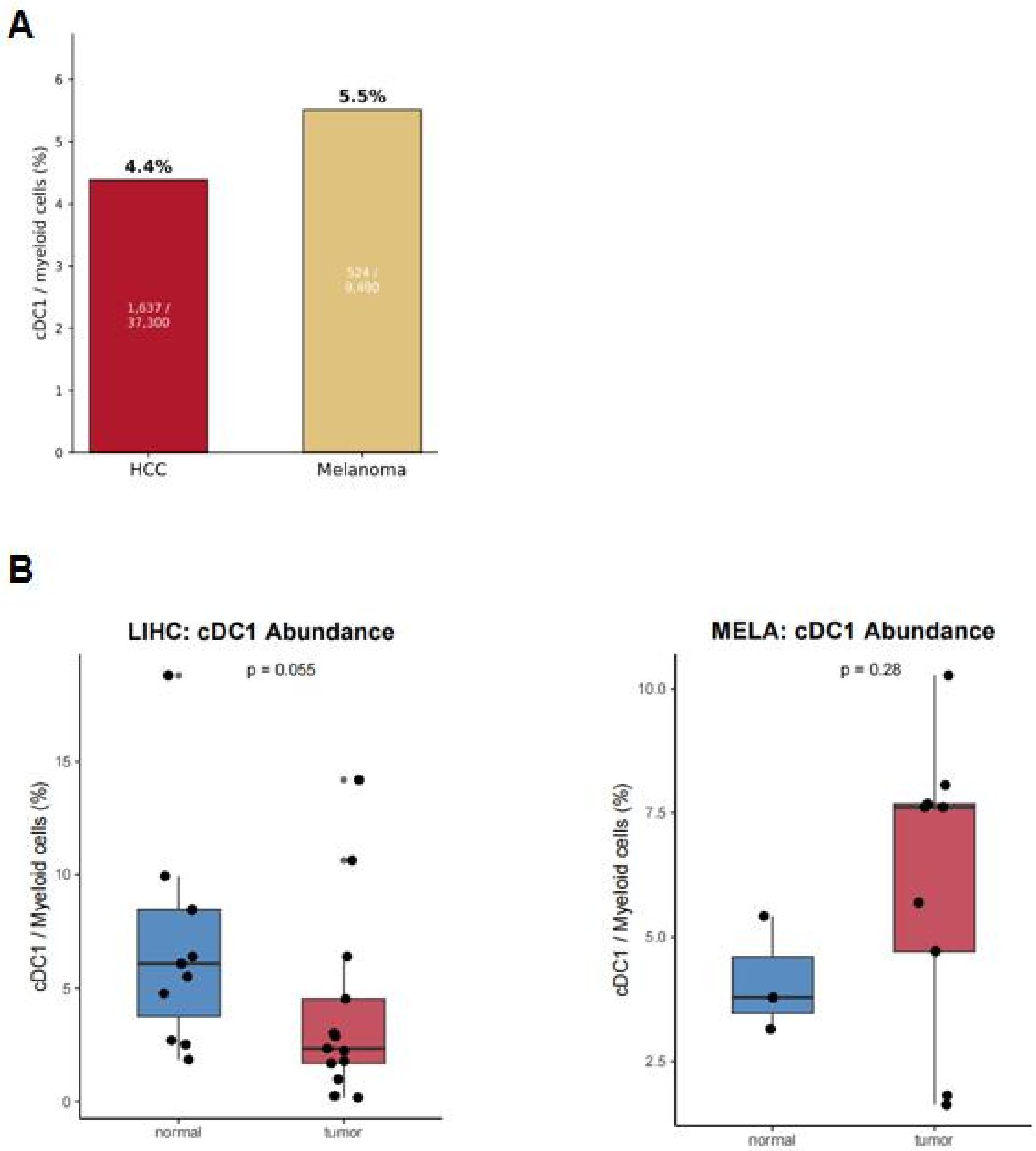

Supplementary Figure 6. cDC1 abundance in different cancer types. (A) Bar plot showing the overall percentage of cDC1 cells in HCC and melanoma scRNA-seq datasets. (B) Box plots showing the proportions of cDC1 cells in normal and tumor tissues from HCC and melanoma cohorts.

Supplementary Figure 7

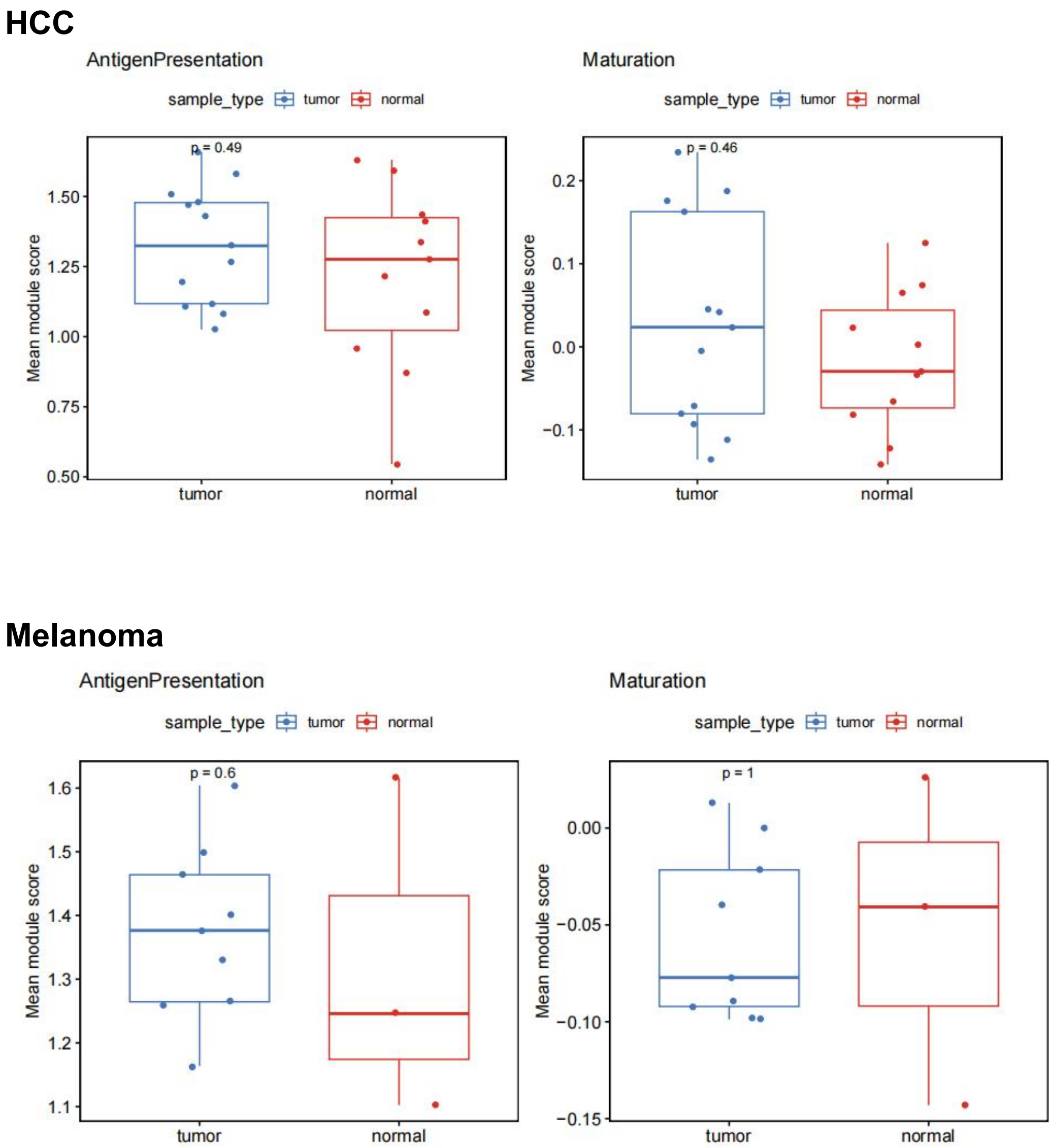

Supplementary Figure 7. Patient-level antigen-presentation and maturation module scores in cDC1.

Supplementary Figure 8

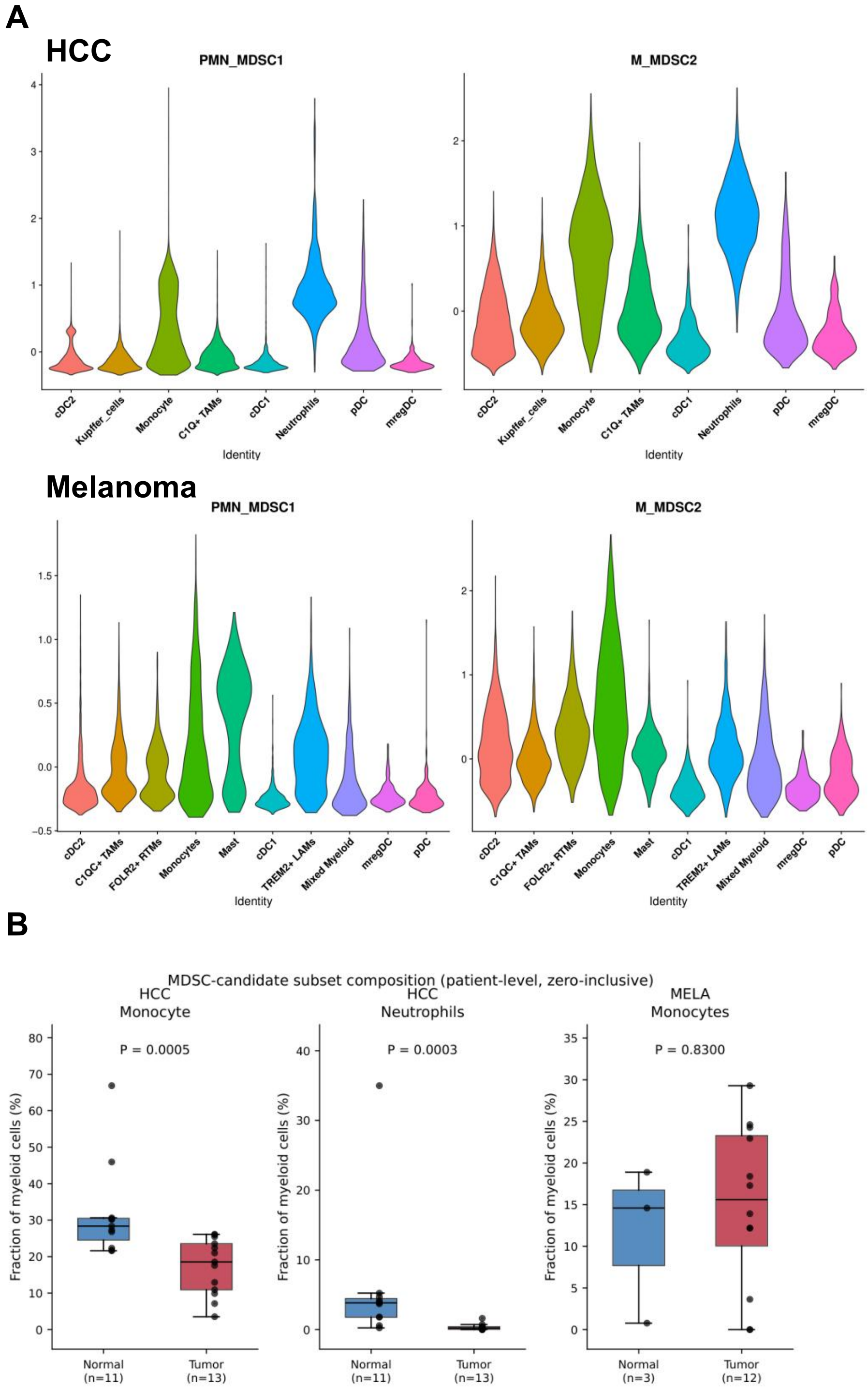

Supplementary Figure 8. MDSC-like suppressive myeloid populations. (A) PMN-MDSC and M-MDSC module scores across myeloid subsets. (B) Patient-level proportions of MDSC-candidate subsets (tumor vs. normal).

Supplementary Figure 9

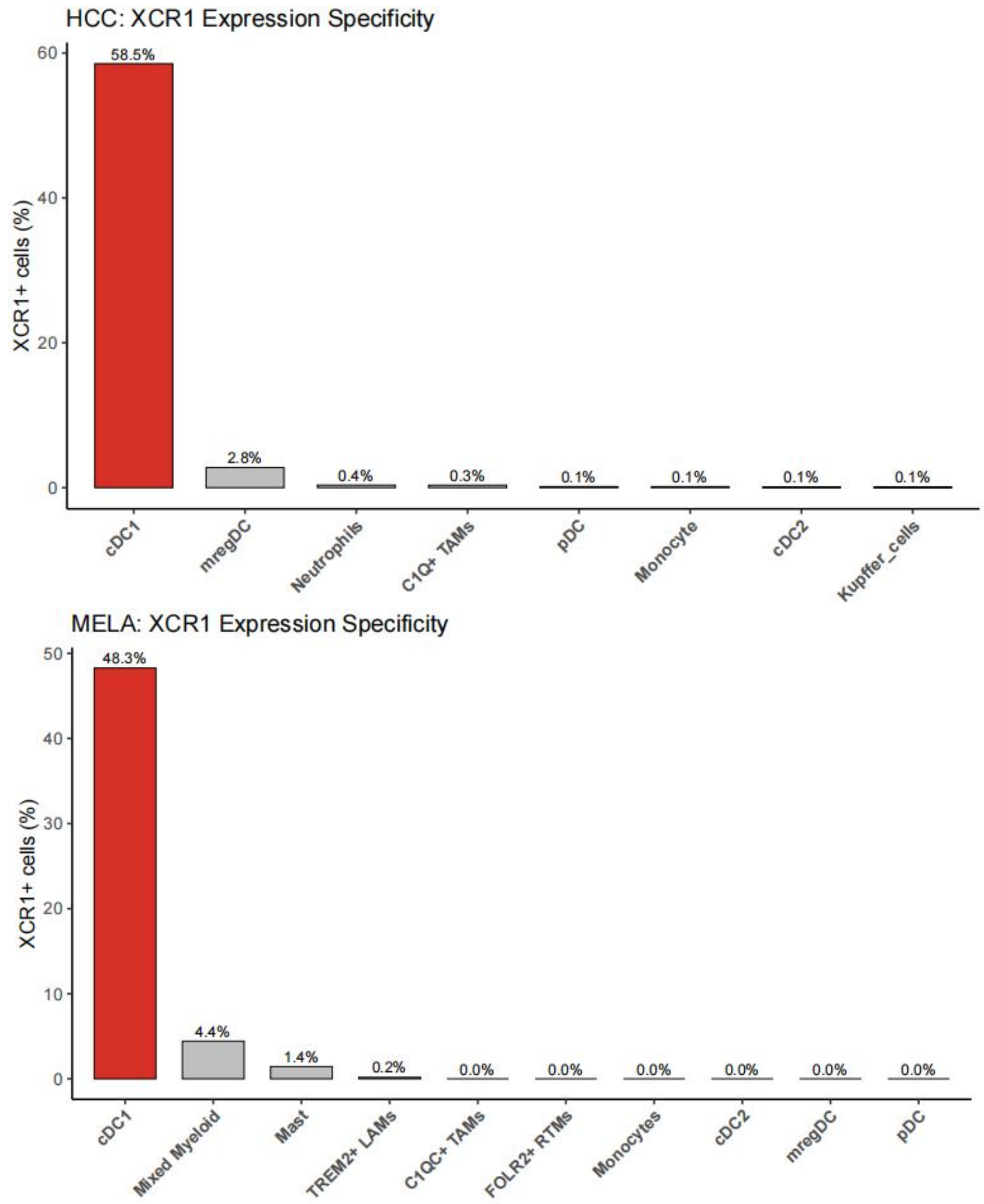

Supplementary Figure 9. XCR1 expression specificity across myeloid cell subsets in HCC and melanoma. Bar charts show the percentage of XCR1 positive cells among distinct myeloid populations from human HCC and melanoma tumor tissues.

Supplementary Figure 10

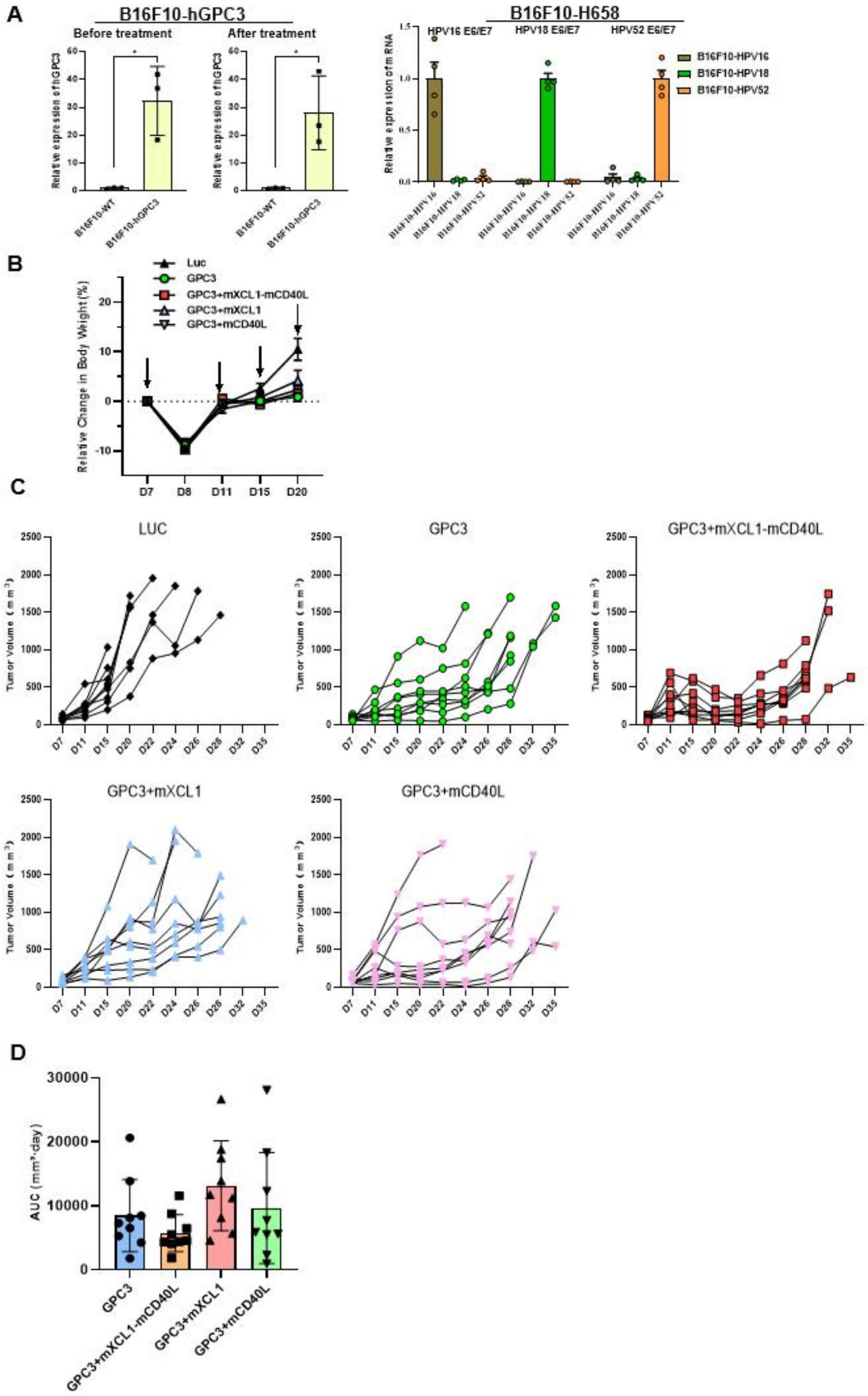

Supplementary Figure 10. Antitumor efficacy of mRNA vaccine combined with mXCL1-mCD40L adjuvant in tumor bearing mice. (A) Characterization of tumor model. qRT-PCR analysis showing relative hGPC3 mRNA expression in B16F10-hGPC3 tumors before and after in-vivo treatment (Left) and B16F10-H658 related cell lines expression (Right). (B) Individual tumor volume growth curves of B16F10-GPC3 tumor bearing mice treated with LUC, GPC3, GPC3+mXCL1-mCD40L, GPC3+mXCL1, or GPC3+mCD40L mRNA-LNP. Each line represents an individual mouse. (D) Quantification of tumor growth area under the curve (AUC, mm³·day) for each treatment group. Each symbol corresponds to one mouse.

Supplementary Figure 11

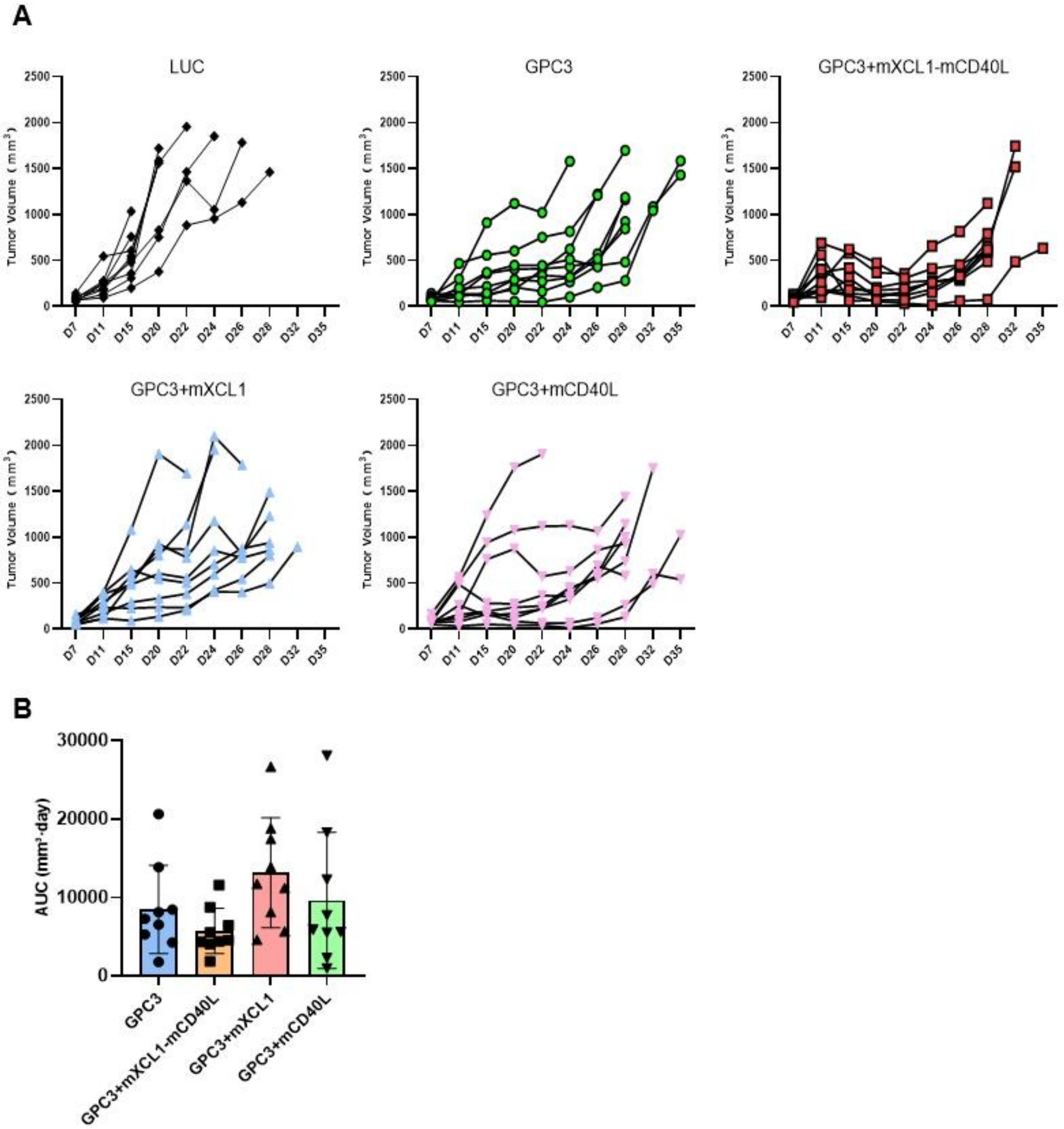

Supplementary Figure 11. Antitumor efficacy of GPC3 vaccine combined with mXCL1-mCD40L adjuvant in tumor bearing mice. (A) Individual tumor volume growth curves of B16F10-GPC3 tumor bearing mice treated with LUC, GPC3, GPC3+mXCL1-mCD40L, GPC3+mXCL1, or GPC3+mCD40L mRNA-LNP. Each line represents an individual mouse. (B) Quantification of tumor growth area under the curve (AUC, $mm^3 \cdot day$) for each treatment group. Each symbol corresponds to one mouse.

Supplementary Figure 12

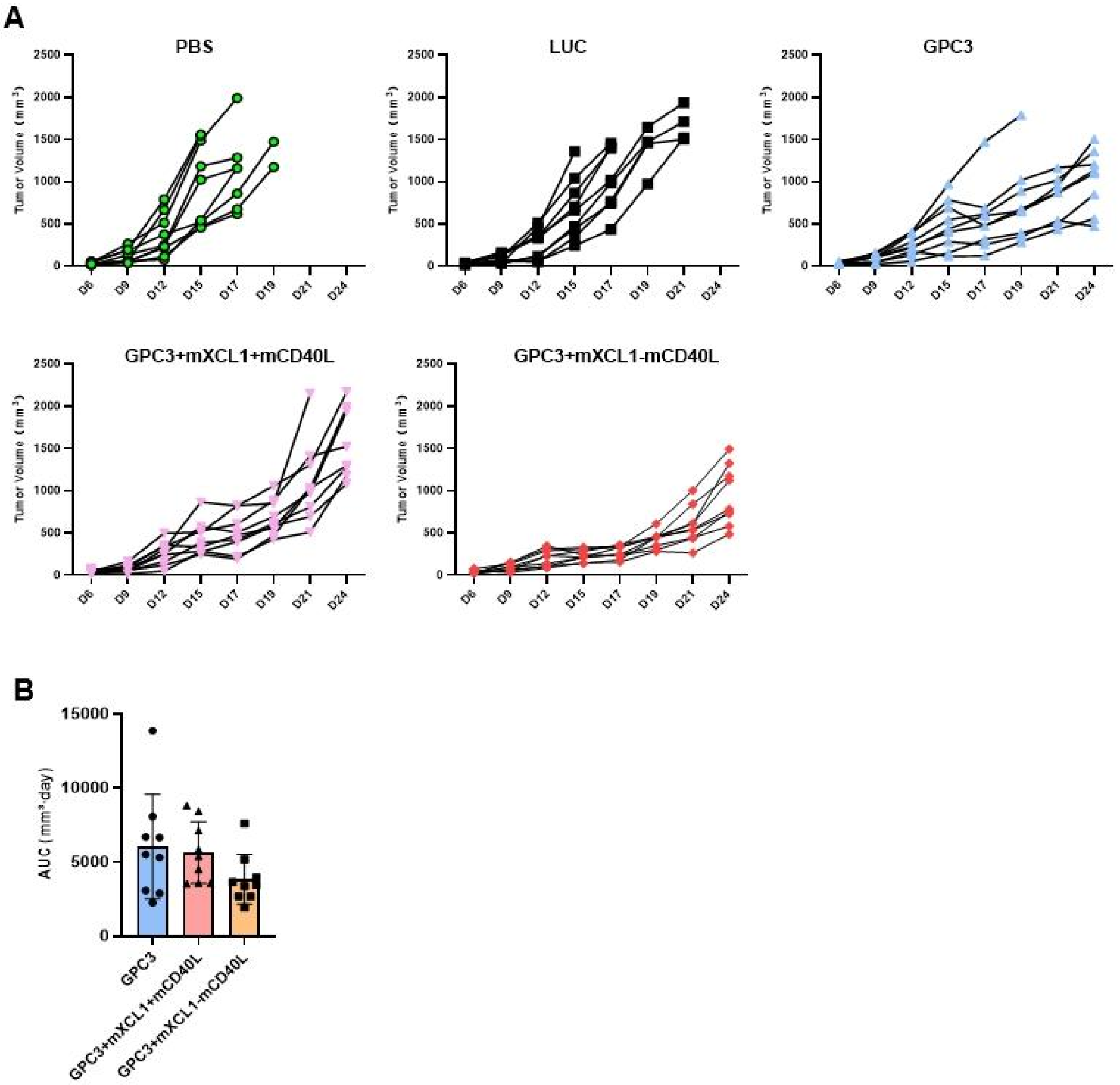

Supplementary Figure 12. Antitumor efficacy of GPC3 vaccine combined with mXCL1-mCD40L fusion adjuvant versus co-delivery of individual adjuvants in tumor bearing mice. (A) Individual tumor volume growth curves of B16F10-GPC3 tumor bearing mice treated with DPBS, LUC, GPC3, GPC3+mXCL1+mCD40L, GPC3+mXCL1-mCD40L mRNA-LNP. Each line represents an individual mouse. (B) Quantification of tumor growth area under the curve (AUC, mm^3·day) for each treatment group. Each symbol corresponds to one mouse.

Supplementary Figure 13

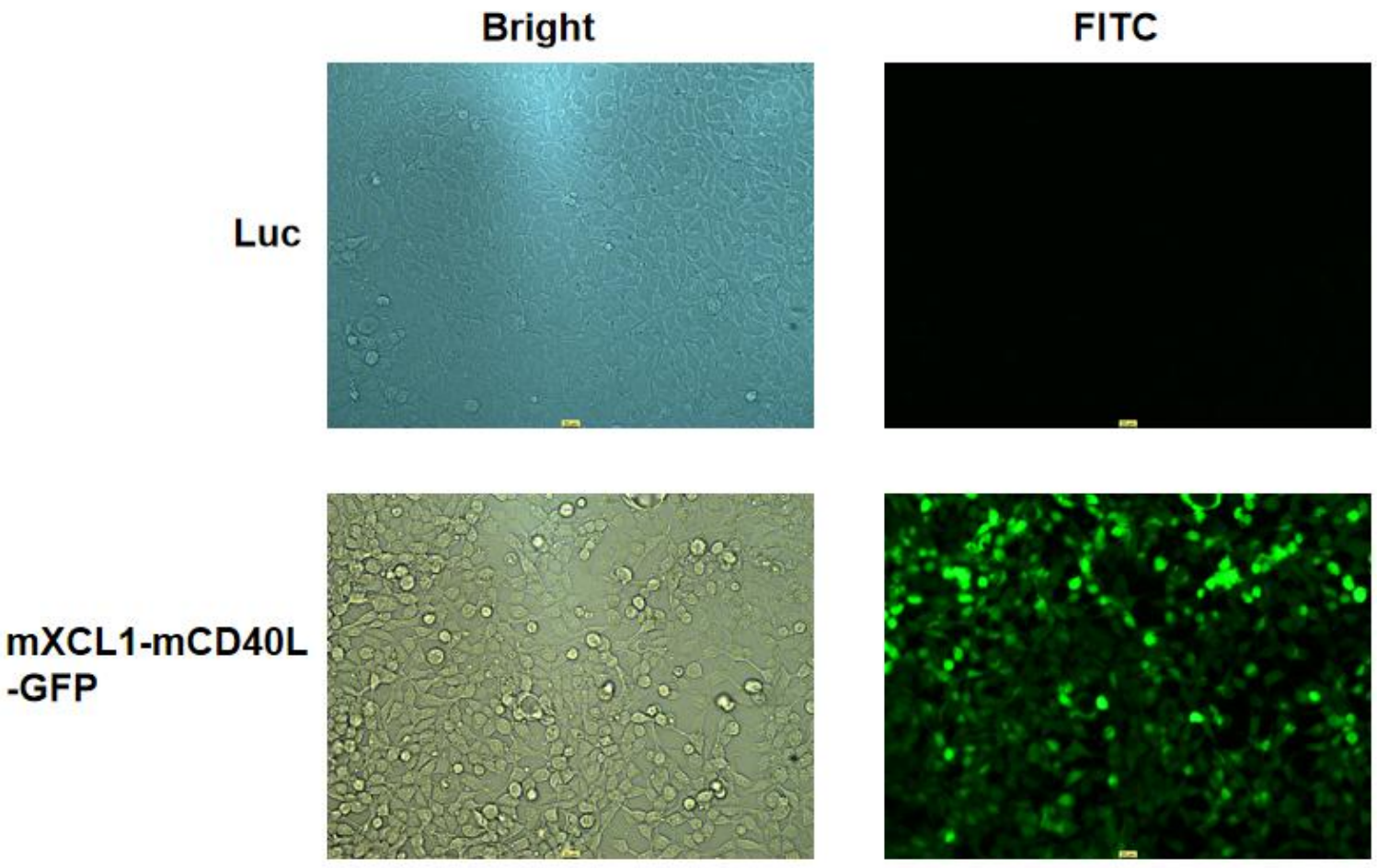

Supplementary Figure 13. GFP fluorescence imaging of mXCL1-mCD40L-GFP expression in transfected B16F10 cells. Bright-field and FITC fluorescence images of B16F10 cells at 24 h after transfection with Luc (control) or mXCL1-mCD40L-GFP mRNA. Strong GFP signal verifies efficient expression of the fusion protein.

Supplementary Figure 14

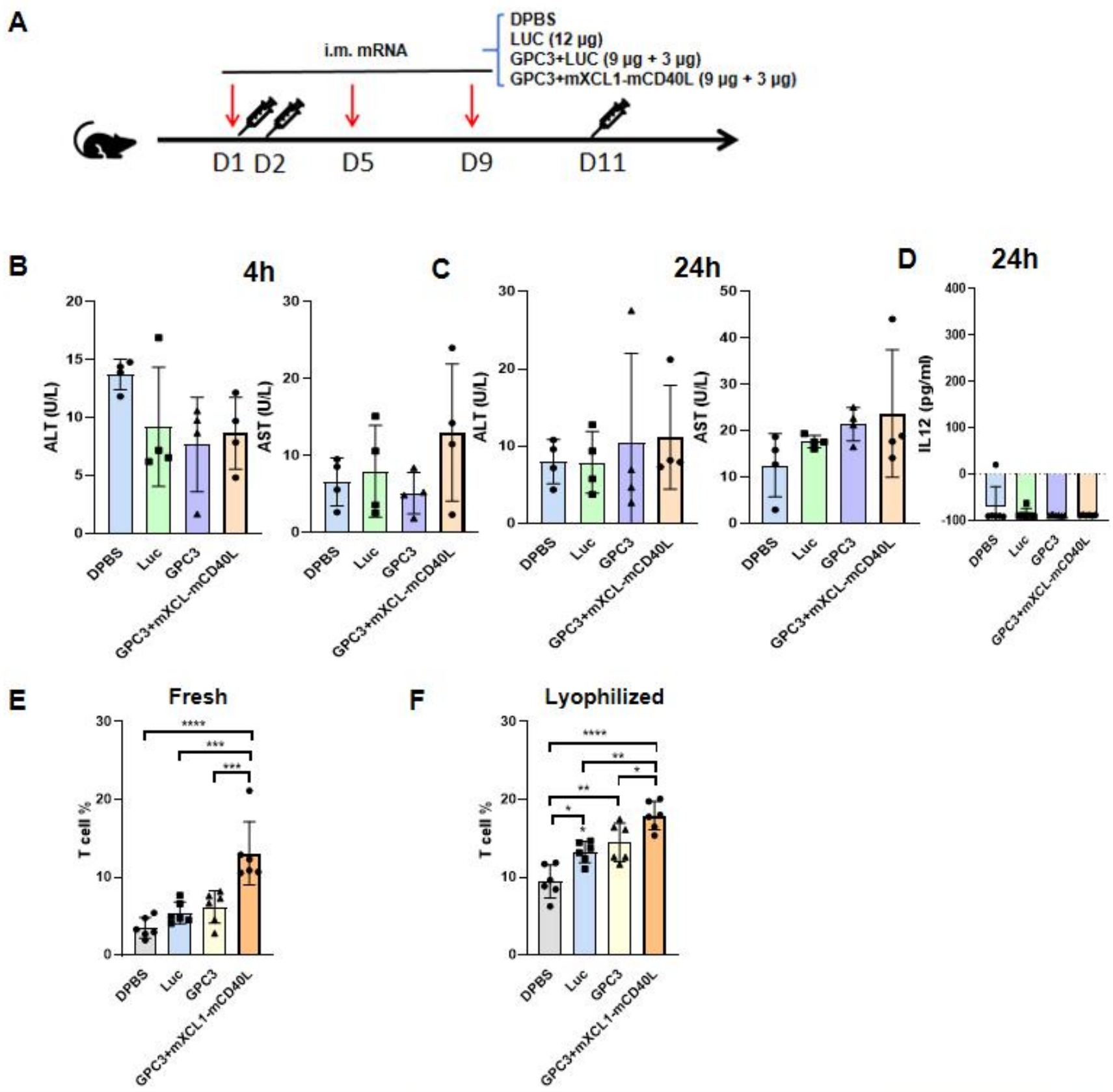

Supplementary Figure 14. Safety evaluation of mXCL1-mCD40L combination therapy and activity validation of the lyophilized formulation. (A) Mouse model construction and administration strategy for safety assessment and immune activation (n=6). (B-C) Serum ALT and AST levels at 4 h and 24 h after the first administration to evaluate hepatotoxicity. (D) Monitoring of serum IL-12 levels 24 h after the initial administration. (E) Flow cytometric results for the proportion of $CD8^+$ T cells in peripheral blood. (F) Validation of activation potency on peripheral blood $CD8^+$ T cells after reconstitution of the lyophilized formulation.
